# Covalent-Organic-Framework-Modified Quartz Crystal Microbalance Sensor for Selective Detection of Hazardous Formic Acid

**DOI:** 10.1021/acsami.4c04630

**Published:** 2024-05-28

**Authors:** Lamiaa Reda Ahmed, Johann Lüder, Cheng-Hsin Chuang, Ahmed F. M. EL-Mahdy

**Affiliations:** † Department of Materials and Optoelectronic Science, National Sun Yat-Sen University, Kaohsiung 80424, Taiwan; ‡ Institute of Medical Science and Technology, National Sun Yat-Sen University, Kaohsiung 804201, Taiwan; § Center for Theoretical and Computational Physics, National Sun Yat-Sen University, Kaohsiung 80424, Taiwan

**Keywords:** covalent organic framework, quartz crystal microbalance, phenylenediamine, formic acid, sensitivity and selectivity

## Abstract

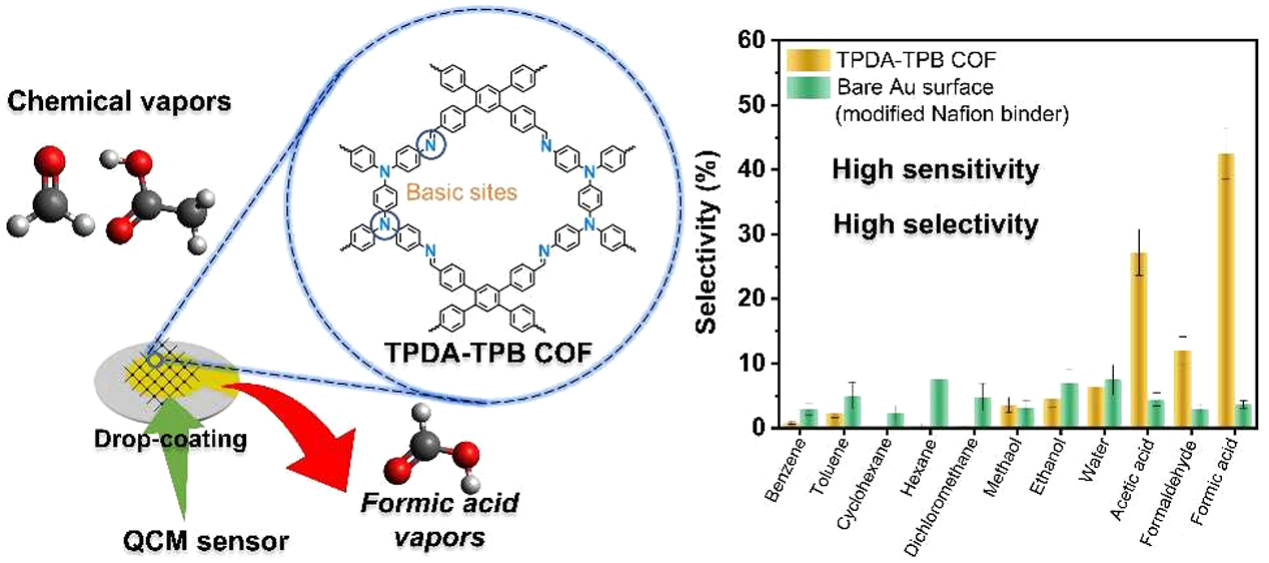

Covalent organic frameworks (COFs) are a novel family of porous crystalline materials utilized in various advanced applications. However, applying COFs as a hazardous organic acid gas sensor is substantial but still challenging. Herein, a phenylenediamine-based covalent organic framework (TPDA-TPB COF) featuring excellent crystallinity, ultrastable thermal stability, and high surface area was successfully constructed. Then, the TPDA-TPB COF-modified quartz crystal microbalance (QCM) sensor is fabricated by immobilizing the TPDA-TPB COF thin film on the gold-QCM chip. The fabricated TPDA-TPB COF-modified QCM sensor demonstrates a rapid response, excellent reproducibility, high selectivity, and sensitivity to formic gas, arising from hydrogen-bonding interactions between formic acid and the outermost layer of the TPDA-TPB COF, as determined by extensive analysis and density functional theory calculations. The basic sites of the TPDA-TPB COF, which are numerous due to its high nitrogen content, and the carboxylic acid groups present in formic acid exhibit efficient interactions. The sensitivity of the TPDA-TPB COF-modified QCM sensor was found to be 7.75 Hz ppm^–1^ at standard room temperature and pressure conditions, with a limit of detection (LOD) of formic acid down to 1.18 ppm, which is significantly below the workplace olfactory threshold limit of 5.0 ppm established by the Occupational Safety and Health Administration. The TPDA-TPB COF-modified QCM sensor exhibits remarkable detecting capabilities, making it highly attractive for detecting organic acid vapors in diverse applications that require superior performance.

## 1 Introduction

Volatile organic compounds (VOCs), which are primary constituents of atmospheric pollution, have the potential to interact with breathed air via processes such as evaporation or sublimation, hence posing risks to both the ecological environment and human health.^1,2^ Hence, a significant area of focus in current research pertains to the development of effective detection methods and removal technologies for VOCs that pose environmental risks. These VOCs include xylene (C_8_H_10_), acetone (CH_3_COCH_3_), formic acid (HCOOH), acetic acid (CH_3_COOH), and formaldehyde (HCHO), as highlighted in previous studies.^3,4^ Among these deleterious VOCs, formic acid is acknowledged as a significant VOC with detrimental effects. It possesses a diverse array of practical uses, such as serving as a preserving or antimicrobial agent. Furthermore, it serves as a precursor in the production of numerous chemicals and materials of higher value. Although highly irritating, harmful, and corrosive, it can be extensively used in leather processing.^5^ Formic acid has been identified to be a promising hydrogen transporter for hydrogen production.^6^ Exposure to formic acid vapor results in exterior chemical burns upon contact with the human body, and inhalation of the vapor can give rise to severe lung disease, dermatosis, and nerve damage.^7^ Furthermore, breathing formic acid can cause various significant illnesses, including bronchial asthma, aberrant immune system functioning, and liver impairment.^8^ Thus, identifying formic acid is critical, and developing a low-cost, immediate sensor for detecting HCOOH in gaseous form might prove valuable for medical investigations and air-quality surveillance. Formic acid is used in various processes, and this apparatus could be used to protect workers and reduce the likelihood of formicary corrosion on machinery and plumbing. Various sensing techniques have been used to build formic acid sensors, including optics, impedance, amperometry, and capacitance.^9−13^ Despite their tremendous power and dependability, these systems are associated with expensive costs, time-consuming processes, the need for highly experienced operators, and large equipment sizes. As a result, there is a major demand for developing novel sensors capable of detecting formic acid with ease, speed, high sensitivity, and selectivity. However, this task presents a considerable obstacle.

Mass-sensing quartz crystal microbalance (QCM) sensors have gained a lot of interest because of their ability to perform real-time monitoring of hazardous gas and/or harmful VOC in a variety of environments.^14,15^ Furthermore, QCM sensors feature fast response times, great sensitivity, and impressive selectivity while remaining user-friendly.^16^ QCMs have exhibited exceptional efficacy across a diverse range of applications, including but not limited to gas sensing, enzyme detection, and polymerization reactions.^17−19^ QCM sensors have been the subject of substantial research in the field of gas sensing, encompassing various gases such as trimethylamine, ammonia gas, mercury vapor, humidity, alcohol, and others.^20−26^ During the operation, it has been shown that the materials coated on the QCM quartz crystal have the ability to absorb gas molecules, resulting in a slight change in mass at the nanogram scale.^27^ The conversion of mechanical energy into electrical energy through the piezoelectric effect enables the realization of sensing by transforming it into a frequency signal. The coating materials on QCM sensors are largely responsible for their degree of sensitivity and selectivity. To date, a limited number of functional materials, including zeolites, polyethylenimine-grafted polyacrylonitrile nanofibers, polysiloxane, carbon nanotubes (CNTs), poly(vinylidene fluoride), polymers, metal–organic frameworks (MOFs), graphene oxide, carbon quantum dots, and hybrid organic/inorganic polymers, have been successfully developed and utilized as sensors for QCM devices.^28−37^ The utilization of QCM sensors for the detection of potent carboxylic acids has been infrequent. As an illustration, Lin and colleagues conducted a study whereby they fabricated a nanocomposite consisting of acidified multiwalled carbon nanotubes (MWCNTs)/polyaniline. This nanocomposite exhibited a notable sensitivity toward formic and acetic acids, with sensitivity values reaching up to 38.51 and 30.70 Hz mg^–1^ m^–3^, respectively.^38^ In their research, Cai et al. generated MWCNTs with an acidic coating. Their results concluded that these nanotubes were extremely sensitive to formic and acetic acids, detecting concentrations as low as 0.77 and 0.73 ppm, respectively.^39^ In addition, Torad and co-workers (2021) produced phenyl-modified carbon nitride quantum nanoflakes to detect formic acid with a sensitivity of 80 ppb.^15^ Because effective and precise detection of organic carboxylic acids requires a large density of binding sites, developing a unique coating material for QCM is necessary.

Crystallized porous organic polymers called covalent organic frameworks (COFs) are distinguished by their powerful covalent bonding that forms periodic prolonged networks. These frameworks exhibit either two-dimensional (2D) or three-dimensional architecture.^40−42^ COFs are distinguished from other porous materials by their unique properties. Their large surface area is partly due to their long-conjugated structures, which also allow a wide variety of organic groups to be functionalized on their surface. COFs also have the capacity to alter their physical characteristics,^43,44^ have uniform micropores, and are very thermally and chemically resilient. Many different fields of study use COFs because of their many useful properties. These include a low mass density, a large specific surface area, and the flexibility to alter their pore size and shape. Applications range from chemical sensors to perovskite solar cells to electrical gadgets to gas and energy storage.^45−51^ Furthermore, it is worth noting that COFs have favorable characteristics for the adsorption of organic molecules due to their notable absorption capabilities and widespread conjugation.^52,53^ The utilization of pure COF as a coating material for VOC identification has been described solely in a report. In their study, He et al. described the synthesis of a benzimidazole-based COF as a coating material for QCM sensors. This COF was utilized for the detection of 2-(chloroethyl)ethyl sulfide (CEES), a volatile organic chemical. According to the cited study, the COF-coated QCM sensor has a sensitivity of up to 0.96 ppm.^54^ However, there is currently no existing literature on the utilization of COFs as coating materials for the purpose of recognizing formic and acetic acids using QCM.

**Figure 1:**
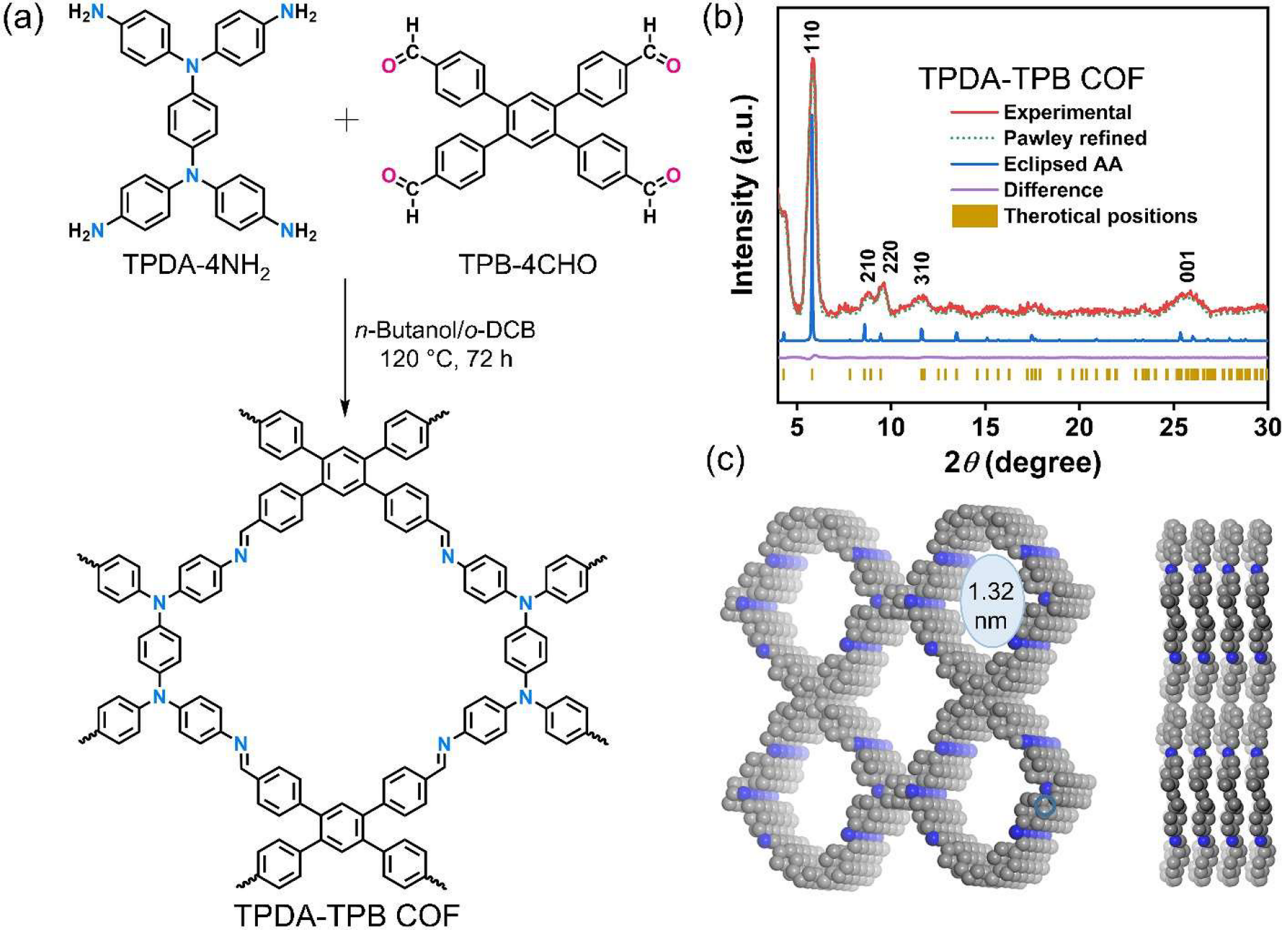
(a) Synthetic route of the TPDA-TPB COF. (b) PXRD patterns of the TPDA-TPB COF: experimental pattern (red), simulated Pawley refined patterns (green), their difference (violet), and simulated patterns obtained from the AA stacking model (blue). (c) Top and side views of the AA-eclipsed model of the TPDA-TPB COF.

In the present study, we report the design and synthesis of a phenylenediamine-based covalent organic framework (TPDA-TPB COF) through polycondensation of *N*^1^,*N*^1′^-(1,4-phenylene)bis[*N*^1^-(4-aminophenyl)benzene-1,4-diamine] (TPDA-4NH_2_) with 4′,5′-bis(4-formylphenyl)-[1,1′:2′,1″-terphenyl]-4,4″-dicarbaldehyde (TPB-4CHO) (Figure 1a). The TPDA-TPB COF exhibits potent crystallinity, long-ordered architecture, significant porosity, and excellent thermal endurance. The TPDA-TPB COF is notable because it has many easily accessible basic nitrogen sites in its periodic channels that could form chemical and hydrogen-bonding interactions with guest organic acid molecules. An intriguing observation is that the TPDA-TPB COF-modified QCM sensor exhibits selectivity toward formic acid compared to formaldehyde, acetic acid, and other interfering gases. The TPDA-TPB COF-modified QCM sensor is distinguished from previous sensors by its exceptional selectivity, sensitivity, and repeatability. The sensor is capable of swiftly detecting formic acid vapor, achieving a lower limit of detection (LOD) of 1.18 ppm at ambient temperature conditions. Furthermore, we employed density functional theory (DFT) calculations to determine the energies and gain insights into the interaction mechanism of formic acid on the exterior surfaces of the TPDA-TPB COF.

## 2 Experimental Section

### 2.1 Materials

Tetrakis(triphenylphosphine)palladium(0) (99%) and palladium on activated carbon (10% Pd/C) were obtained from Acros. 1,2,4,5-Tetrabromobenzene (97.0%) and 4-formylphenylboronic acid (95.0%) were purchased from Sigma-Aldrich. Hydrazine monohydrate (≥98%), *p*-phenylenediamine (97%), and 1-fluoro-4-nitrobenzene (99%) were obtained from Alfa Aesar.

### 2.2 Synthesis of the TPDA-TPB COF

TPDA-4NH_2_ (100 mg, 0.21 mmol) and TPB-4CHO (104.64 mg, 0.21 mmol) were placed in a 25 mL tube. They were then mixed with a solution containing equal parts *o*-dichlorobenzene and *n*-butanol (1:1, 10 mL), as well as a 6 M aqueous solution of acetic acid (1.0 mL). The tube underwent multiple freeze/pump/thaw cycles to remove gas and was subsequently flame-sealed and then heated to a temperature of 120 °C. Following a period of 72 h, a solid substance was produced. This solid was then subjected to filtration and underwent many rounds of washing using acetone and tetrahydrofuran in a sequential manner. A 91% yield of the TPDA-TPB COF was achieved using vacuum drying at 120 °C for 24 h.

### 2.3 Materials Characterization

The Fourier transform infrared (FTIR) spectra were obtained by utilizing a Bruker Tensor 27 FTIR spectrophotometer and a traditional KBr plate technique. A total of 32 scans were conducted at a resolution of 4 cm^–1^. A Bruker Avance 400 NMR spectrometer was used to record solid-state NMR spectra. The technique of cross-polarization with magic angle spinning was employed to obtain ^13^C NMR spectral data at a frequency of 75.5 MHz. A Siemens D5000 instrument was utilized to perform powder X-ray diffraction (PXRD) analysis. The X-ray source used was monochromated Cu Kα (λ = 0.1542 nm). Transmission electron microscopy (TEM) analysis was conducted using a JEOL-2100 scanning electron microscope. Field-emission scanning electron microscopy (FE-SEM), specifically using a JEOL JSM-7610F model, was utilized to perform the observation. Prior to the observation, the COF material underwent a process of platinum sputtering for a duration of 100 s. The produced samples, weighing around 50–100 mg, were analyzed using a BELSORP MAX analyzer to assess their Brunauer–Emmett–Teller (BET) surface area and porosity. Thermogravimetric analysis (TGA) was conducted using a TA Q-50 analyzer in the presence of a nitrogen (N_2_) environment. The specimens were enclosed within a platinum cell and subjected to a temperature increase from 40 to 800 °C at a rate of 20 °C min^–1^ while being exposed to a flow of N_2_ gas at a rate of 50 mL min^–1^. The process of molecular modeling was carried out using *Reflex*, a software program designed for crystal determination based on X-ray diffraction (XRD) patterns. This software was applied in MS modeling, version 4.4, developed by Accelrys. The unit cell dimensions were first established manually by utilizing the coordinates of the observed XRD peak locations.

### 2.4 Fabrication of a TPDA-TPB COF-Modified QCM Electrode and Its QCM Sensor

An AT-cut gold-coated quartz crystal electrode with a thickness of 300 nm, a size of 7.9 × 7.9 mm, and a fundamental frequency of 8.94 MHz ± 30 kHz (QA-A9M Au, SEIKO EG&G Co. Ltd., Japan) was used as a gold-coated QCM electrode, and a QCM922A quartz crystal microbalance (QA-A9M Au, SEIKO EG&G Co. Ltd., Japan) was used as a frequency counter. The gold-coated QCM electrode was immersed in a combination of ethanol and water (in a ratio of 3:1) and subjected to ultrasonic waves for a duration of 30 min. Subsequently, the electrode was dried in a vacuum oven at a temperature of 60 °C. Then, the gold-coated QCM electrode was connected inside a 1.5 L testing gas sensor vessel,^14,15^ and the fan was turned on. We subsequently opened all valves to purge N_2_ gas at a flow rate of 100 mL min^–1^. Then, its fundamental QCM frequency (*F*_0_) was measured to be 8943218 Hz.

In order to apply a layer of TPDA-TPB COF to the gold-coated QCM electrode, a uniform mixture of the COF sample (2.0 mg) and Nafion binder (1.0 mL/0.05 wt %) in aqueous solution was first prepared and subjected to sonication for a duration of 30 min. Then, the TPDA-TPB COF-modified QCM electrode was fabricated by depositing a 5 μL droplet of yjr TPDA-TPB COF sample over the top layer of the gold-coated QCM electrode at room temperature. The TPDA-TPB COF-modified QCM electrode was allowed to remain uninterrupted for a period of 30 min. The electrode was subsequently dried overnight inside a convection oven under vacuum at 60 °C.

The TPDA-TPB COF-modified electrode was connected inside the 1.5 L testing gas sensor vessel,^14,15^ and the fan was turned on. We subsequently opened all valves to purge N_2_ gas at a flow rate of 100 mL min^–1^ inside the vessel until a stable frequency line (±1 Hz min^–1^) was obtained. Then, the frequency was recorded after coating as *F*_1_, which was found to be 8946485 Hz. Subsequently, *F*_0_ and *F*_1_ were utilized to determine the mass of the COF sample deposited on the gold-coated QCM electrode, employing the Sauerbrey equation (eqs 2 and 3). The mass of the TPDA-TPB COF sample coated on the gold-coated QCM electrode was computed to be 3.48 μg.

### 2.5 QCM Sensor Experiments

QCM sensor experiments were done using the TPDA-TPB COF-modified QCM sensor.^14,15^ During each vapor exposure, the N_2_ gas flow was halted, the air became the background gas, the fan was turned on, and the temperature and relative humidity within the glass vessel were recorded as 25 ± 2 °C and 47 ± 3%, respectively. Afterward, a gaseous form of the analytes was obtained at ambient temperature (25 ± 2 °C) by the injection of analytes in liquid form with known volume inside the testing gas sensor vessel by a Hamilton microliter syringe (Hamilton Company Inc., Switzerland).^14,15^ Parts per million (ppm) of vapor concentration were determined using the following method (eq 1, which takes into account both the density and mass concentration):
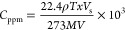
1The variables *C*_ppm_, *V*, *T*, ρ, *V*_s_, *x*, and, M respectively represent the concentration of vapors, the working glass vessel volume (L), the cell temperature (K), the density of the injected liquid analyte (g mL^–1^), the volume of the injected chemical analyte (μL), the purity of the analyte, and the molecular weight of the injected chemical analyte (g).

The gas-sensing capabilities of the TPDA-TPB COF toward formic acid (HCOOH) vapors were examined alongside vaporized substances, including benzene, toluene, cyclohexane, hexane, dichloromethane, methanol, ethanol, water, acetic acid, and formaldehyde in the static sensing system by recording the frequency change (Δ*F*, Hz) of the QCM caused by vapor species additional mass at 25 ± 2 °C. Computers equipped with the *WinQCM* software assessment automatically captured the time-dependent frequency when the liquid analyte was injected sequentially.

The TPDA-TPB COF-modified QCM electrode, permeated with vapor analyte, was subjected to a flow of N_2_ at a rate of 2 L min^–1^ to remove the chemical analyte molecule until a consistent frequency variation was attained. When the electrode returned to the initial frequency, it signified that the chemical-vapor analyte had effectively desorbed.

The QCM sensor technology can accurately detect the shift in the oscillation frequency due to the increased mass of adsorbed vapor particles at the nanogram level. Sauerbrey explored the relationship between the change in frequency (Δ*F*, Hz) and the mass per unit area (Δ*m*, g cm^–2^) accumulated on the gold electrode of the QCM sensor at the initial resonant frequency (*F*_0_) using eqs 2 and 3.
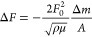
2
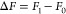
3The variables ρ, *F*_0_, *A*, and μ respectively represent the density of quartz (2.649 g cm^–3^), the initial resonance frequency of the crystal (Hz), the electrode surface area (5 mm diameter, 0.196 cm^2^), and the elastic shear modulus (2.947 × 10^11^ g cm^–1^ s^–2^). All of the recorded frequencies were normalized by mass to determine the sensor sensitivity and selectivity.

The surface area, porosity, and shape of COF materials regulate the adsorption rate of HCOOH vapor, which may be affected by their structural features. Thus, the process of HCOOH adsorption can be thought of as a pseudo-first-order mass transfer from the vapor phase to the COF material. This may be studied by monitoring the frequency just after injection of HCOOH into the glass container. The experimental Δ*F* values of the QCM sensor were fit using a pseudo-first-order kinetic model. Equations 4–6 were used to get the pseudo-first-order kinetic rate constant (*k*_1_), which represents the initial rate of HCOOH vapor uptake (Δ*F*_*t*_/Δ*F*_∞_).
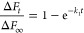
4
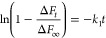
5
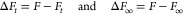
6where Δ*F*_*t*_ and Δ*F*_∞_ represent the vapor uptake frequency changes at time *t* and equilibrium, respectively. The pseudo-first-order kinetic model adsorption rate constant is *k*_1_ (min^–1^). At *t* = 0, *F* represents the oscillating crystal frequency before analyte exposure, whereas *F*_*t*_ and *F*_∞_ are the crystal frequencies following injection at time *t* and equilibrium, respectively.

## 3 Results and Discussion

### 3.1 Construction and Spectral Analysis of the TPDA-TPB COF

The organic crystallite of the TPDA-TPB COF was synthesized solvothermally through a Schiff base condensation reaction of TPDA-4NH_2_ (Scheme S1 and Figures S1–S3) with TPB-4CHO (Scheme S2 and Figures S4–S6). The reaction was conducted using a mixture of AcOH (6 M, 10 vol %) in *o*-dibromobenzene and *n*-BuOH in a 1:1 (v/v) ratio at a temperature of 120 °C for a duration of 3 days, as depicted in Figure 1a. The crystallinity of the produced TPDA-TPB COF was analyzed using PXRD. Parts b and c of Figure 1 show the results of the PXRD analysis, which showed that the TPDA-TPB COF had a microcrystalline arrangement characterized by a long-ordered architecture. The PXRD pattern obtained from the experiment (Figure 1b, red curve) exhibited a prominent and well-defined peak at a 2θ value of 5.86°, which we ascribe to the 110 reflections. Additionally, we observed smaller peaks at 2θ values of 8.67°, 9.56°, and 11.55°, which we credit to the 210, 220, and 310 reflections, respectively. The broad diffraction signal observed at a 2θ value of 25.59° can be attributed to the 001 reflection. This reflection arises due to the presence of strong π-stacking interactions between the tetragonal 2D interlayers of the TPDA-TPB COF. By employing the Bragg equation, we have estimated the interlayer distance between the layers of TPDA-TPB COF. Additionally, we have determined the *d* spacings between the 110 reflections (*d*_110_) of the TPDA-TPB COF to be 3.47 Å and 1.50 nm, respectively. These values are presented in Table S1. The *Materials Studio* software was employed to optimize the potential 2D packing configurations of the TPDA-TPB COF to assess its crystalline framework architecture. The blue line in Figure 1b shows that the observed positions of the experimental peaks in PXRD analysis of the TPDA-TPB COF agreed with the positions predicted by the model based on the AA-eclipsed stacking structure. This finding validates the tendency of the COF to have a strong affinity for highly eclipsed π stacking. The experimental patterns (Figure 1b, red curve) agreed with the simulated PXRD patterns of the TPDA-TPB COF (Figure 1b, green curve) with only very minor discrepancies (Figure 1b, violet curve). In addition, the simulated data of the TPDA-TPB COF provided weighted residual error (*R*_wp_) and residual error (*R*_p_) values of 9.43% and 6.52%, respectively. (Table S2). In order to conduct a more comprehensive analysis of the conformations and unit cell characteristics of the TPDA-TPB COF that we synthesized, we have acquired the following values for the unit cell of the AA stacking model: *a* = 20.60 Å, *b* = 22.60 Å, *c* = 3.51 Å, α = 90°, α = 90°, and γ = 90° (Figures 1c and S7 and outlined in Table S2).

**Figure 2:**
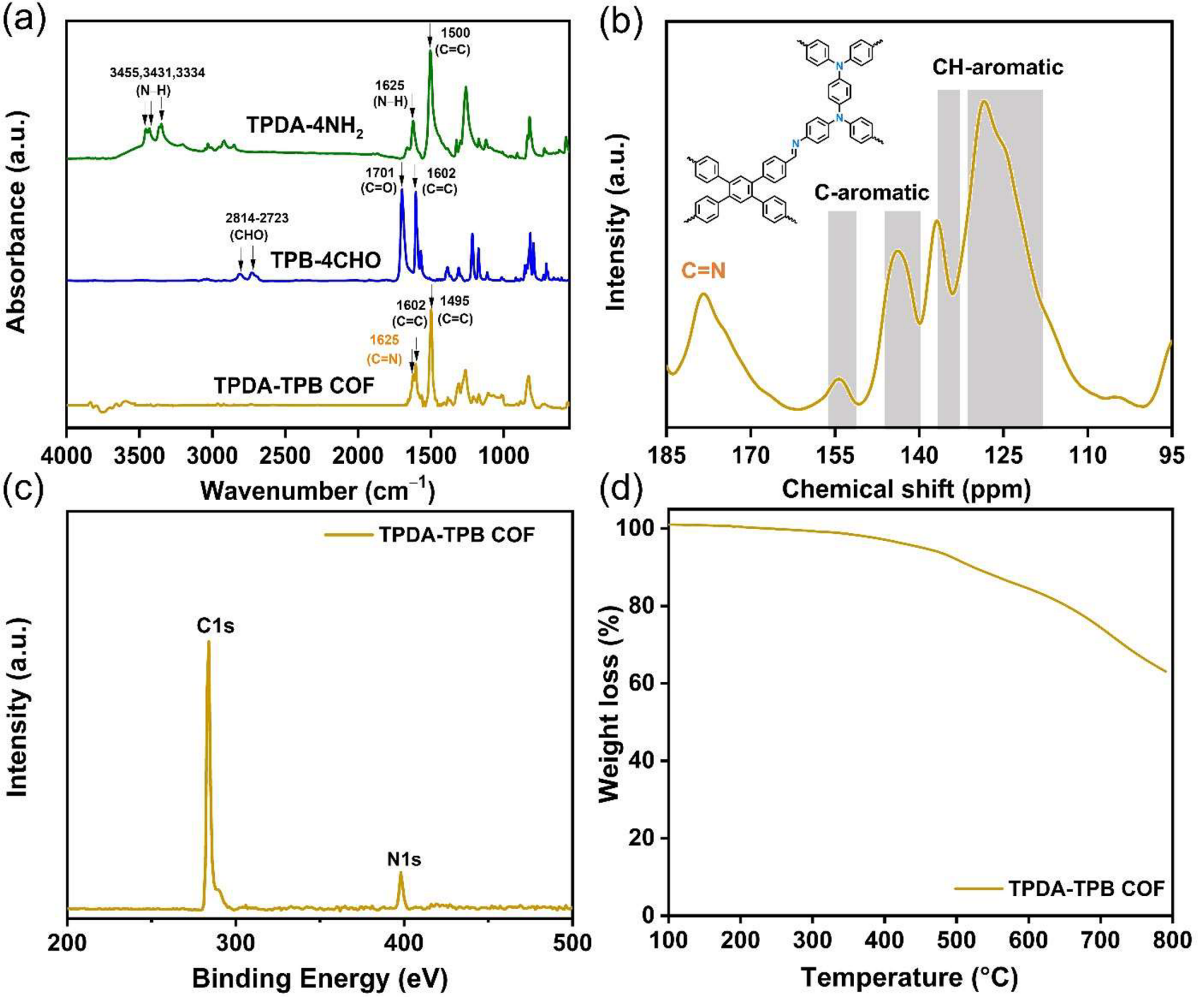
(a) FTIR spectra of TPDA-4NH_2_, TPB-4CHO, and the TPDA-TPB COF. (b) Solid-state ^13^C NMR spectrum of the TPDA-TPB COF. (c) XPS survey spectrum of the TPDA-TPB COF. (d) TGA analysis of the TPDA-TPB COF.

The utilization of FTIR and solid-state ^13^C NMR spectroscopy techniques examined the chemical constituent of the TPDA-TPB COF. Figure 2a shows the FTIR spectrum of the TPDA-TPB COF along with a comparison of the spectra of the monomers to show the increase and tumble of the peaks. The TPDA-4NH_2_ compound displayed prominent peaks at 3455 and 3431 cm^–1^, suggesting an equal and strong splitting of the NH_2_ antisymmetric stretching mode due to Fermi resonance. The presence of the symmetric stretching mode results in the recognition of an additional NH stretching mode at a frequency of 3334 cm^–1^ (Figure 2a). Additionally, strong absorptions around 1500 and 1625 cm^–1^ were seen for the stretching C=C groups and the bending amine N–H groups. The FTIR spectra of TPB-4CHO exhibited distinct peaks at wavenumbers ranging from 2814 to 2723 cm^–1^ for the aldehydic CH=O groups, at 1701 cm^–1^ for the aldehydic C=O groups, and at 1602 cm^–1^ for the C=C groups (Figure 2a). The FTIR spectra of the TPDA-TPB COF exhibited an absence of any discernible signals corresponding to the N–H groups of TPDA-4NH_2_ and the aldehydic CH=O and C=O groups of TPB-4CHO. This absence suggests that these functional groups have undergone complete consumption. The FTIR spectrum of the TPDA-TPB COF (Figure 2a) exhibited a characteristic absorption peak at a wavenumber of 1625 cm^–1^, corresponding to the stretching vibrations of the imino C=N functional groups. Furthermore, the TPDA-TPB COF displayed two distinct bands of absorbance around 1602 and 1495 cm^–1^, indicating the existence of aromatic C=C group stretching (Figure 2a). Figure 2b presents the solid-state ^13^C NMR spectrum of the TPDA-TPB COF. For compression, the ^13^C NMR spectrum of TPDA-4NH_2_ displayed a peak at 144.85 ppm corresponding to the carbon atoms bonded to amino groups (C–NH_2_) (Figure S3). Conversely, the ^13^C NMR spectrum of TPB-4CHO revealed a peak at 192.47 ppm corresponding to the carbon atoms in the aldehydic (CH=O) functional group (Figure S6). These signals are strongly decreased after the polycondensation of TPDA-4NH_2_ with the TPB-4CHO. Furthermore, the solid-state ^13^C NMR spectrum (Figure 2b) of the TPDA-TPB COF exhibited a peak at 178.43 ppm corresponding to the imino C=N carbon nuclei and peaks ranging from 159.81 to 109.20 ppm corresponding to the aromatic carbon nuclei. The observed spectra are in agreement with the transformation of the initial substances into the imine-based COF. X-ray photoelectron spectroscopy (XPS) was utilized to assess the elemental chemical states present in the TPDA-TPB COF. The data presented in Figure 2c demonstrate the presence of two clearly distinguishable peaks corresponding to the C 1s and N 1s orbitals at energy levels of 284.40 and 398.35 eV, respectively, in the TPDA-TPB COF. Figure 2c also shows that the lack of any additional components discovered by XPS supports the conclusion that no noticeable impurities were produced during the TPDA-TPB COF synthesis. The ability of porous polymers to endure elevated temperatures is of utmost importance for their prospective utilization in sensors and commercial applications. Hence, an investigation was carried out to assess the thermal stability of the TPDA-TPB COF through the implementation of TGA within a temperature range of 100–800 °C under a N_2_-laden environment (Figures 2d and S8). The TPDA-TPB COF revealed an early weight loss of roughly 4.89% before 455 °C, which can be attributed to the desorption of trapped solvents. Furthermore, the TPDA-TPB COF exhibited significant thermal stability, as indicated by its decomposition with two distinct peaks at temperatures of 500 and 709 °C. This suggests that the network backbone decomposed gradually (Figure S8). According to the data presented in Figure 2d, it can also be observed that the TPDA-TPB COF experienced a weight drop of 10% when subjected to a temperature of 523 °C. Additionally, a char yield of 63.12% was obtained under these conditions. Furthermore, the covalently connected crystalline network arrangement of the TPDA-TPB COF led to its excellent thermal stability, with a total weight loss of around 36.88%.^55^

**Figure 3:**
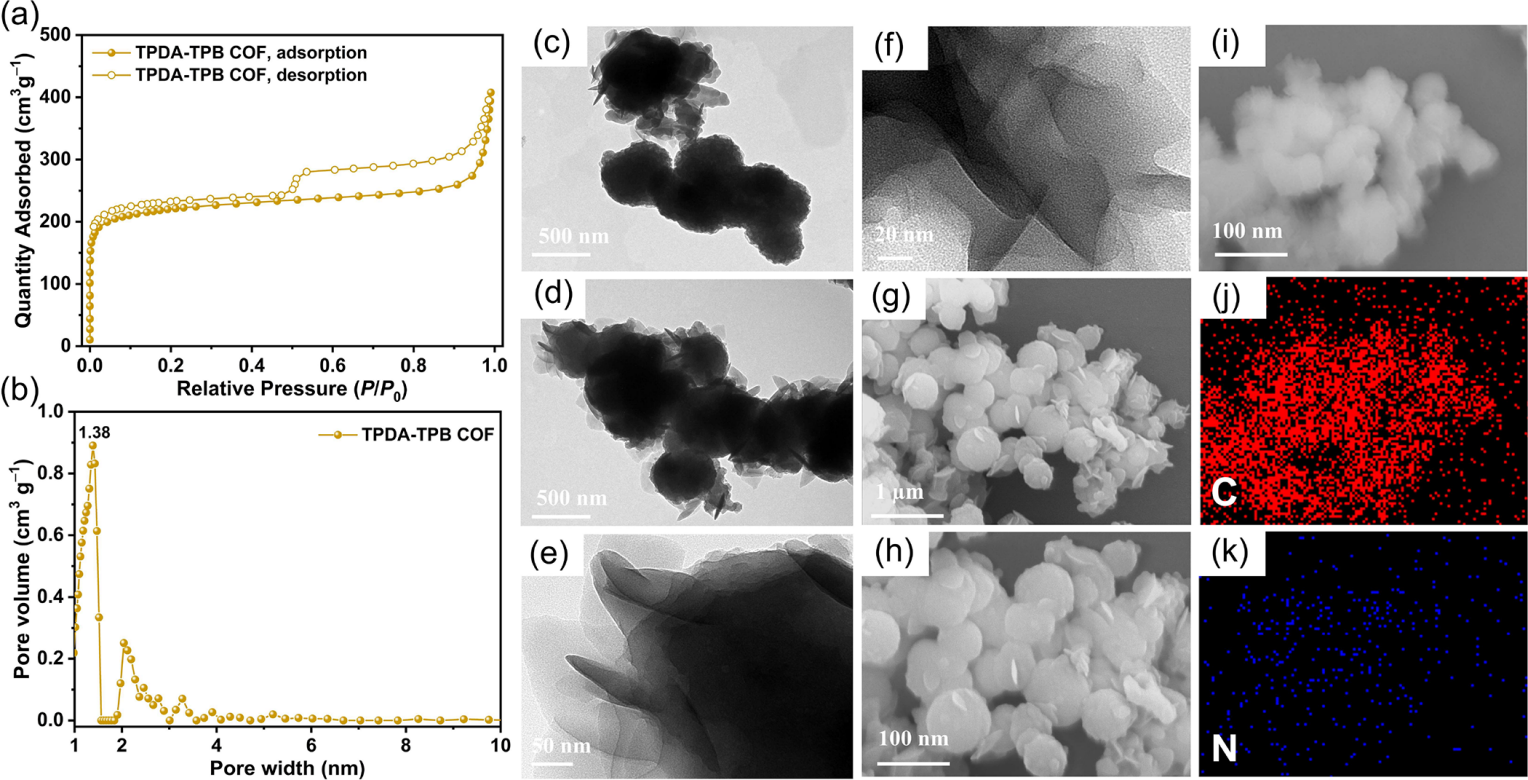
(a) N_2_ sorption isotherm of the TPDA-TPB COF at 77 K. (b) Pore-size distribution of the TPDA-TPB COF. TEM images of the TPDA-TPB COF recorded at high magnification with scale bars of (c and d) 500 and (e) 50 nm and (f) at low magnification with a scale bar of 20 nm. FE-SEM images of the TPDA-TPB COF recorded at magnification with scale bars of (g) 1 μm and (h and i) 100 nm. (j and k) EDS elemental mapping images of the TPDA-TPB COF.

We recorded the N_2_ adsorption/desorption isotherm of our synthesized TPDA-TPB COF at a temperature of 77 K for assessing the porosities of its framework (Figure 3a). The investigation of the sorption curve exhibited a type I isotherm characterized by pronounced increases at low-pressure levels of *P*/*P*_0_ < 0.01, which suggests the existence of persistent micropores. The surface area and pore volume of the TPDA-TPB COF were examined using the BET model. The results revealed that the TPDA-TPB COF exhibited a surface area of 852 m^2^ g^–1^ (Figure S9) and a pore volume of 0.89 cm^3^ g^–1^, as presented in Table S1. Additionally, the microporous architecture of the TPDA-TPB COF was elucidated through analysis of the pore-size distribution profile using nonlocal DFT. Figure 3b and Table S1 demonstrate that the TPDA-TPB COF possessed a pore size of 1.38 nm. This is consistent with the theoretical pore size provided by the crystal structures, which averaged 1.32 nm (Figure 1c). The morphology of the TPDA-TPB COF was analyzed using TEM and FE-SEM. TEM imaging revealed that the TPDA-TPB COF exhibited a compact arrangement, forming spherical nanostructures with a diameter ranging from 400 to 450 nm (Figure 3c,d). The shape and structure of the resulting COF have been shown to be considerably influenced by the degree of planarity of the building component.^56,57^ Additionally, it has been observed that the utilization of nonplanar building monomers in the construction of COFs often leads to the production of spherical structures.^48,56^ Hence, it is hypothesized that the lack of planarity in the TPDA-4NH_2_ and TPB-4CHO building monomers has played a role in the development of the spherical morphology observed in the TPDA-TPB COF. The high-resolution TEM pictures at reduced magnification provided visual evidence of the porosity characteristics of the TPDA-TPB COF, as depicted in Figure 3e,f. Furthermore, verification of the spherical morphology of the TPDA-TPB COF was conducted through imaging using FE-SEM (Figure 3g–i). Further, it was observed that carbon and nitrogen atoms exhibited a homogeneous distribution across the frameworks of both TPDA-TPB COFs. This was confirmed through elemental mapping analysis conducted using energy-dispersive X-ray spectroscopy (EDS; Figure 3j,k).

### 3.2 COF-Based QCM Sensor for Organic Acids

**Figure 4:**
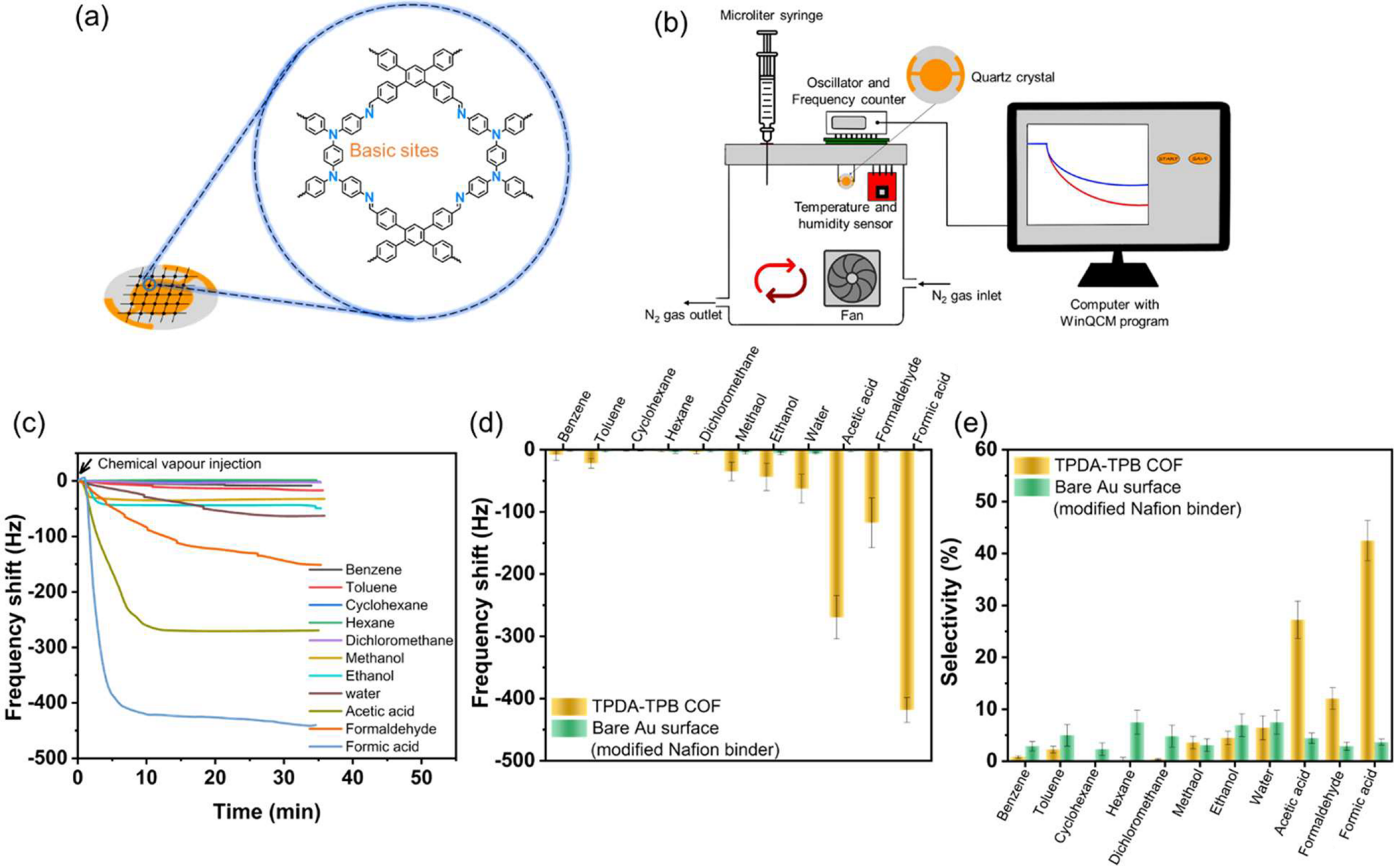
(a) Fabrication of a film of the TPDA-TPB COF on the gold electrode of a QCM. (b) Schematic representation of the TPDA-TPB COF-modified QCM sensor measuring setup. (c) Mass-normalized time-dependent frequency changes upon adsorption of different VOCs and water (53 ppm) by the TPDA-TPB COF-modified QCM sensor at the temperature and relative humidity within the glass vessel of 25 ± 2 °C and 47 ± 3%. (d) Summary of the mass-normalized frequency variations recorded by the TPDA-TPB COF-modified QCM sensor following being subjected to a wide range of VOCs and water (53 ppm). (e) Selectivity values for various vapors at the temperature and relative humidity within the glass vessel of 25 ± 2 °C and 47 ± 3% for the TPDA-TPB COF-modified QCM sensor. Subsequent to the organic VOCs being injected at a concentration of 53 ppm, the frequencies were recorded.

Formic acid, the carboxylic acid with the lowest molecular weight, is characterized by its extreme volatility, intense pungency, caustic nature, lack of color, corrosiveness, and flammability. Additionally, it is a poisonous gas with a terrible odor. Prolonged and excessive exposure to a certain formic acid can lead to many adverse effects, such as inflammation, damage to the nerves, and even loss of vision.^58^ In this study, we introduce a highly effective QCM-modified chemical sensor with exceptional sensitivity in detecting small concentrations of formic acid. The synthesized TPDA-TPB COF exhibits a significant quantity of active-base sites and possesses a well-organized structure, which holds great potential for applications in formic acid-based sensing. To showcase the applicability of the TPDA-TPB COF, we proceeded to create a TPDA-TPB COF-modified QCM to detect dangerous formic acid gas (Figure 4a,b). According to early data, the TPDA-TPB COF crystallites include active sites and increase the adsorption of HCOOH gas (Figure S10). However, it should be noted that the unadorned gold surface of the QCM electrode, when treated with a Nafion binder, lacks the necessary sensitivity to accurately detect the chemical-vapor analyte (Figure S10).

Selectivity is a critical aspect in determining the effectiveness of the COF-coated QCM sensor. Our synthetically modified TPDA-TPB COF-modified QCM electrode was subjected to formic acid gas (53 ppm), as shown in Figure 4c,d, and the time-dependent frequency shift (Δ*F*) of the QCM sensor was recorded. Formic acid causes the greatest adsorption uptake of Δ*F* = 418.40 ± 20 Hz, which is significantly greater than that of various other chemical vapors (all at 53 ppm), namely, benzene (8.02 ± 9 Hz), toluene (21.89 ± 8 Hz), cyclohexane (1.45 ± 1 Hz), hexane (2.18 ± 1 Hz), dichloromethane (3.86 ± 3 Hz), methanol (35.03 ± 15 Hz), ethanol (43.79 ± 22 Hz), water (62.77 ± 23 Hz), acetic acid (269.36 ± 35 Hz), and formaldehyde (117.52 ± 40 Hz), indicating its exceptional sensitivity toward acidic substances (Figure 4c,d). In addition, it is seen that the TPDA-TPB COF crystallites experience a significant decrease in frequency upon initial adsorption. The occurrence of this phenomenon might be ascribed to the significant assimilation of acidic molecules on the vast surface area of the COF film, along with the existence of multiple basic nitrogen sites within the film. The observation above demonstrates the impact of high crystallinity and a well-organized structure on improving sensitivity in detecting and quantifying a specific guest molecule.

The injection of formic acid results in a significant and noticeable reduction in the frequency of quartz crystals compared to formaldehyde. The decrease can be ascribed to the higher polarity of formic acid, which results in stronger interactions, such as hydrogen bonding and polar–polar, with the nitrogen centers of the TPDA-TPB COF. In contrast, when exposed to formic acid vapor, the observed change in frequency (Δ*F*) of the TPDA-TPB COF-modified QCM sensor is roughly twice as high as that for acetic acid. This suggests a higher level of sensitivity in quantifying formic acid. The higher permittivity of formic acid compared to acetic acid can account for this phenomenon.^59^ A weaker QCM response of acetic acid is anticipated because formic acid (p*K*_a_ = 3.75) is more acidic, and acetic acid (p*K*_a_ = 4.75) is less acidic.^60^ The exceptional sensing capabilities and preferential absorption of acidic chemicals, as opposed to other chemical-vapor analytical substances, can be attributed to the highly ordered crystalline structure of the TPDA-TPB COF. This structure provides ample exposed surface area, allowing for effective interactions between the basic sites of the TPDA-TPB COF (which are abundant due to its high nitrogen content) and the carboxylic acid groups present in formic acid. Additionally, it should be noted that formic acid exhibits reducing properties and can transfer electron density to the TPDA-TPB COF.

In control experiments that include the injection of various chemical gases at a concentration of 53 ppm, the resulting Δ*F* values for each gas were recorded and are presented as follows: benzene (1.5 ± 1 Hz), toluene (2.6 ± 1.3 Hz), cyclohexane (1.2 ± 1 Hz), hexane (3.9 ± 2 Hz), dichloromethane (2.5 ± 1.6 Hz), methanol (4.6 ± 2.3 Hz), ethanol (5.6 ± 2.8 Hz), water (5.9 ± 1.4 Hz), acetic acid (2.3 ± 1.2 Hz), formaldehyde (1.5 ± 1.9 Hz), and formic acid (1.9 ± 0.6 Hz) (Figure 4d). The adsorption capabilities exhibited by the whole surface of Nafion are notably inferior to those of the TPDA-TPB COF material due to Nafion’s small surface area and lack of binding sites for engaging with chemical-vapor analytical substances.

The estimation of the selectivity of the TPDA-TPB COF toward the analyte of gaseous formic acid was conducted using eq 7.^15^
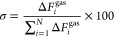
7The sensor response value of the QCM to a specific gaseous analyte is indicated as . The selectivity values (σ) are graphed against several types of chemical-vapor analytical substances at a concentration of 53 ppm, resulting in the generation of Figure 4e. The results of the experiment indicate that the TPDA-TPB COF-modified QCM sensor exhibits a significant level of selectivity toward formic acid vapor compared to other chemical-vapor analytical substances. At a concentration of 53 ppm, the TPDA-TPB COF-modified QCM sensor shows a selectivity of around 42.47 ± 3.9% toward formic acid vapor, which is notably higher than the selectivity observed for other chemical-vapor analytes.

The presence of humidity might lead to a shift in selectivity during the detection of volatile organic molecules. Hence, it is imperative to consider the impact of humidity on the selectivity of formic acid in the design of a sensor intended for deployment in indoor settings such as residential or occupational spaces. Consequently, conducting an interference test becomes important to assess the performance of the sensor accurately. The sensing response of the TPDA-TPB COF-modified QCM sensor against formic acid vapor was evaluated and compared to its response toward water vapor, both at a concentration of 53 ppm. This comparison is shown in Figure 4d,e. The sensor has a response to formic acid (Δ*F* = 418.40 ± 20 Hz) that is about 7 times greater than its response to water vapor (Δ*F* = 62.77 ± 23 Hz). This indicates that the sensor is well-suited for the selective detection of formic acid vapor in a humid environment. In addition, the TPDA-TPB COF exhibits a selectivity of about 6.43 ± 2.3% toward water vapor. Furthermore, we examined how the TPDA-TPB COF-modified QCM sensor responded to formic acid vapor at various relative humidity levels. Figure S11 demonstrates that the TPDA-TPB COF has a good selectivity for formic acid vapor even in conditions of high humidity. The obtained outcome suggests that, although an increased water content in the experimental container may elicit a reaction, any resultant signal interference is considerably less compared to the response induced by formic acid. The remarkable sensing capabilities exhibited by the TPDA-TPB COF indicate that the process of water absorption in this material is likely governed by a kind of physical adsorption characterized by low binding strength.

**Figure 5:**
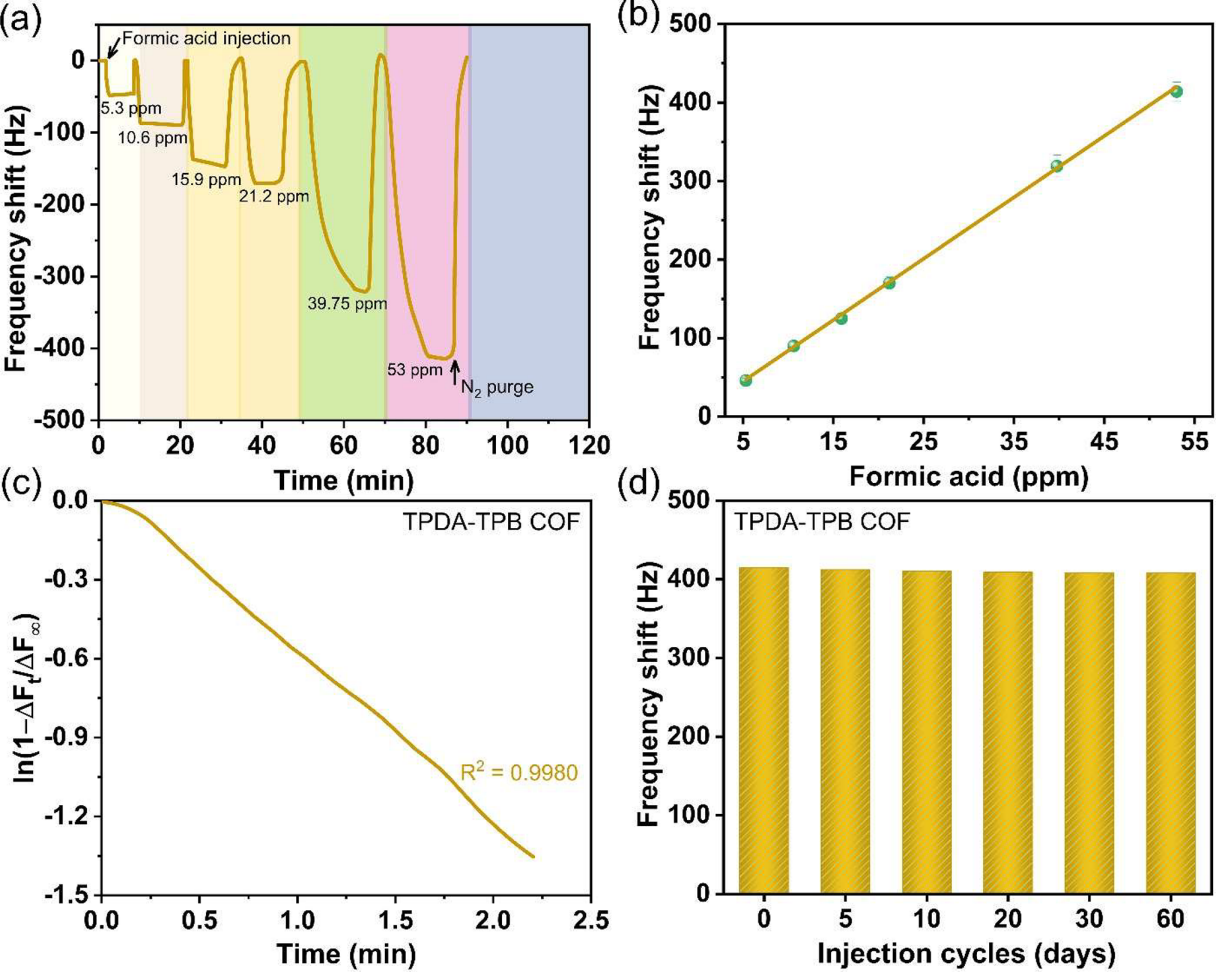
(a) Mass-normalized time-dependent frequency shift of the TPDA-TPB COF-modified QCM sensor when subjected to various formic acid concentrations at the temperature and relative humidity within the glass vessel of 25 ± 2 °C and 47 ± 3%. (b) Linear fitting of formic acid vapor concentrations by the TPDA-TPB COF-modified QCM sensor. Each measurement was performed three times. (c) Plot of ln(1 – Δ*F*_*t*_/Δ*F*_∞_) against time *t* for the TPDA-TPB COF-modified QCM sensor. (d) Stability of the TPDA-TPB COF-modified QCM sensor upon injection of formic acid vapor (53 ppm) over a period of 2 months.

Figure 5a depicts the real-time measurement of the QCM-modified TPDA-TPB COF sensor for various amounts of formic acid vapor, ranging from 5.3 to 53 ppm. The TPDA-TPB COF-modified QCM sensor demonstrates the capability to detect formic acid vapor even at low concentrations of 5.3 ppm, resulting in a frequency change (Δ*F*) of roughly 46 Hz. The detection effectiveness of formic acid vapor is notably enhanced with increasing concentrations of injected formic acid. This results in a frequency shift of 418.40 Hz at a concentration of 53 ppm (Figure 5a). According to the calibration graph presented in Figure 5b, it can be observed that the sensing responses of the TPDA-TPB COF-modified QCM sensor exhibit a linear rise when the concentration of injected formic acid increases. The system sensitivity enables the effective use of the TPDA-TPB COF for detecting small amounts of vapor-phase HCOOH, achieving an impressively low LOD of 1.18 ppm when standard ambient temperature and pressure settings are used. The LOD is determined by employing the calibration curve derived from the data presented in Figure 5b. The equation used for this calculation was *y* = 7.75*x* + 5.27, with an *R*^2^ value of 0.9993, as described in eq 8.
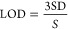
8The response’s standard deviation is denoted as SD, while the calibration curve’s slope is represented as *S*. The lower LOD of the TPDA-TPB COF-modified QCM sensor for HCOOH vapor is significantly below the workplace olfactory threshold limit of 5.0 ppm established by the Occupational Safety and Health Administration (Table S3). This threshold represents the concentration at which vapor-phase formic acid develops, harming the nose, throat, and eyes of particularly susceptible individuals.^58^ The TPDA-TPB COF exhibits a more effective sensing capability for formic acid in comparison to previously documented substances, such as amperometric biosensors,^11^ impedance-based sensors,^10,13^ MOFs, polymeric-material-based QCM sensors,^38,61,63^ carbon nitrides,^62^ MWCNTs,^39^ chemiresistors,^60^ and fluorescence sensors^63^ (Table S4).

The study focused on examining the adsorption kinetics of formic acid by the real-time monitoring of changes in the frequency shift (Δ*F*) of a TPDA-TPB COF-modified QCM sensor. Subsequently, the initial rate of formic acid vapor adsorption was analyzed using a pseudo-first-order kinetic model. The kinetic study was performed and evaluated utilizing eq 5. Figure 5c exhibits linear regression on the plots of ln(1 − Δ*F*_*t*_/Δ*F*_∞_) over time *t* with a correlation coefficient (*R*^2^) of 0.9980, confirming the pseudo-first-order kinetic model of the adsorption of formic acid over the TPDA-TPB COF-modified QCM sensor. The rate constant (*k*_1_) of the pseudo-first-order kinetic model for the TPDA-TPB COF was determined to be 5.92 × 10^–2^ min^–1^ (Figure 5c). Furthermore, the pseudo-first-order reaction kinetics of formic acid adsorption on the TPDA-TPB COF-modified QCM sensor verified that the adsorption rate is only influenced by the formic acid concentration. The air concentration is adequately elevated, leading to an insignificant alteration in its concentration.

Additionally, an examination was conducted on the enduring stability of the TPDA-TPB COF-modified QCM sensor, a crucial aspect pertaining to gas sensor materials and devices (Figure 5d). The long-term stability of the TPDA-TPB COF-modified QCM sensor’s detecting response to a concentration of 53 ppm formic acid was investigated over a duration of 2 months, demonstrating exceptional performance. It is worth noting that the data also indicate a decline of just 6.59% in the Δ*F* value of the TPDA-TPB COF-modified QCM sensor when formic acid is continuously injected. This observation highlights the remarkable long-term stability and outstanding cycling efficiency of the sensor. Moreover, the chemical stability of the TPDA-TPB COF was examined over 2 months of use as a sensing material for detecting 53 ppm formic acid. This was done by conducting measurements utilizing FTIR and TEM. Figure S12 demonstrates that the FTIR peaks detected in the TPDA-TPB COF showed no statistically significant changes. Furthermore, the TEM pictures showed that the TPDA-TPB COF retained its spherical shape even after being exposed to formic acid vapor for a long period (Figure S13). The results demonstrate the exceptional stability of our TPDA-TPB COF.

### 3.3 DFT Calculations of Organic Acid Adsorption at the Surface of a COF

To obtain an understanding of the system at the atomic scale, we performed DFT calculations with the *Gaussian16* package^64^ at the B3LYP^65−67^ level of theory, including dispersion corrections with the DFT-D3^68^ method with a 6-31G basis set.^69^ We modeled the interaction between gas-phase molecules (adsorbate) and segments of the COF (adsorbent) representing one full macrocycle, i.e., a monomer unit. Details about the monomer unit are given in Figure S14. Complete structure optimization was performed for all structures. The interaction strength is taken as the adsorption energy (*E*_ad_) and calculated according to , where  is the total energy of the COF with attached molecules and *E*_COF_ and *E*_Mol_ are the respective total energies of the COF and adsorbate.

Because the adsorption of adsorbate on the COF showed many local minima, we sampled at least 40 nonequivalent adsorption sites. The initial adsorption sites and adsorbate orientations were selected randomly with distances between 0.32 and 0.36 nm. Our calculations result in different interaction strengths between adsorbates and the COF. Formic acid has the strongest exothermic binding of −0.880 eV, followed by acetic acid (−0.848 eV) and formaldehyde (−0.378 eV). Note that basis set superposition-error-corrected complexation energies obtained with methods by Boys and Bernardi^70^ showed a similar trend with −0.843, −0.816, and −0.238 eV.

**Figure 6:**
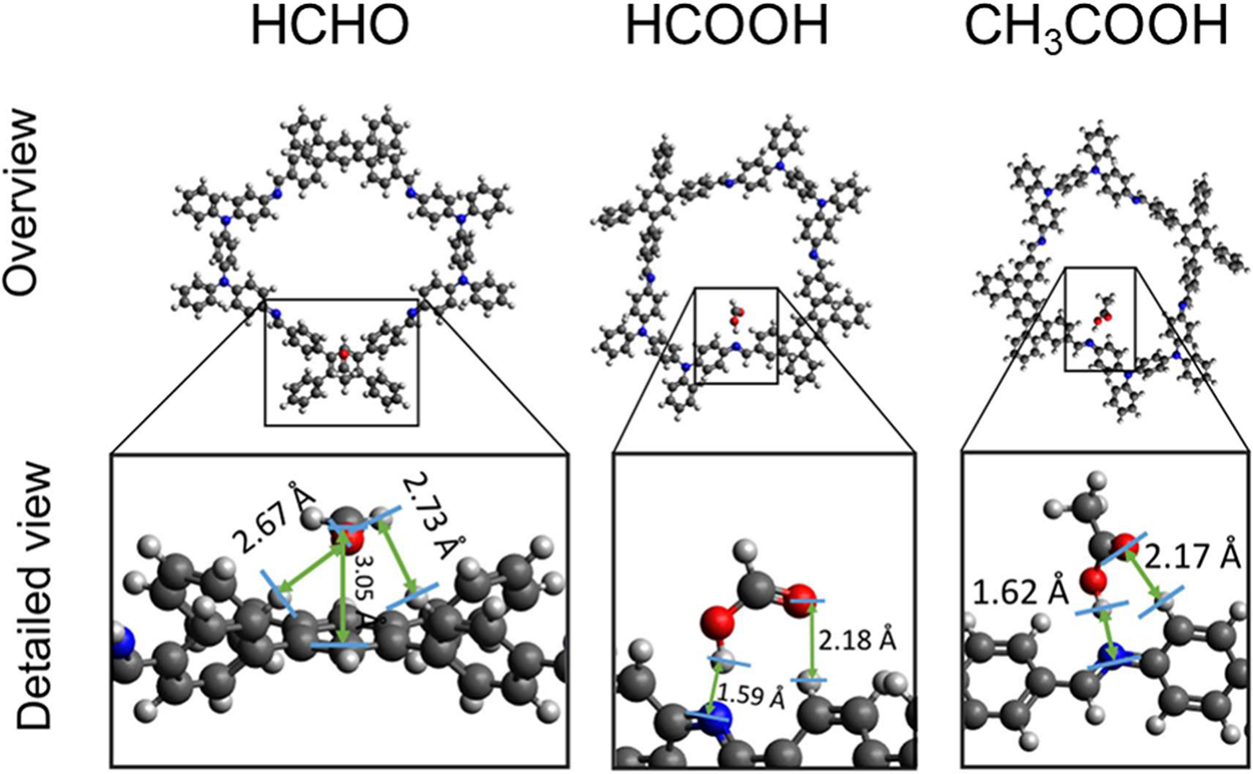
Structural information on HCHO, HCOOH, and CH_3_COOH adsorbed on a computational COF model. The top panel provides an overview, and the bottom panel provides detailed views of the adsorption site and binding distances.

Details about the structure of adsorbates on the COF are given in Figure 6. The adsorbates have adsorption distances between 0.159 and 0.267 nm that inversely correlate with the binding strength; i.e., formic acid (HCOOH) has the shortest distance and strongest binding. In contrast, formaldehyde (HCHO) has the largest distance and weakest binding. In general, van der Waals and hydrogen bonds seem to drive binding to the COF. HCHO adsorbs parallel to a benzene-like ring with a distance of about 0.3 nm. We observed shorter distances between the hydrogen atoms of HCHO and hydrogen atoms from neighboring benzene rings of the COF. CH_3_COOH binds similarly to HCOOH at the amine/imino group of the COF. For the latter two molecules, binding occurs between the OH group of the adsorbates and the nitrogen atom in the COF with distances of about 0.16 nm and between the oxygen atom of the adsorbates and a CH group of the adsorbent with distances of about 0.22 nm. Hence, CH_3_COOH and HCOOH both take advantage of forming two connections to the COF simultaneously due to their structural similarity in the vicinity of their carboxyl group. Among the experimentally tested adsorbates, only CH_3_COOH and HCOOH have carboxyl groups that can use this effect. This correlates with the sensor’s performance, which is superior for these molecules over all other experimentally tested adsorbates. Last, the stronger binding with HCOOH supports the experimentally observed increased selectivity for this molecule.

## 4 Conclusions

In conclusion, we synthesized the TPDA-TPB COF with excellent crystallinity, ultrastable thermal stability, and high surface area. Due to its remarkable nanostructure properties and long-ordered structure, the TPDA-TPB COF was used to construct an effective QCM sensor. By employment of the TPDA-TPB COF, it became possible to achieve enhanced sensitivity in detecting formic acid vapor while also improving the ability to discriminate against other interfering vapor-phase analytes, all under ambient conditions of temperature and pressure. The TPDA-TPB COF-modified QCM sensor exhibited a sensitivity of 7.75 Hz ppm^–1^ under standard room temperature and pressure settings. The LOD for formic acid was determined to be 1.18 ppm. DFT calculations revealed the interaction mechanism between formic acid and the TPDA-TPB COF and evidenced the selectivity of the sensor toward carboxyl-containing compounds. The results of these calculations suggest that both chemical interactions and hydrogen bonding play a role in the interactions between formic acid and the TPDA-TPB COF. This study has the potential to facilitate the advancement of effective gas-sensing technology.

## Supporting Information Available

Syntheses of TPDA-4NH_2_, TPB-4CHO, and the TPDA-TPB COF, FTIR and ^1^H and ^13^C NMR spectra of TPDA-4NH_2_ and TPB-4CHO, PXRD and BET parameters of the synthesized TPDA-TPB COF, crystalline structure for the TPDA-TPB COF based on an AA-eclipsed stacking model along the *c* and *a* axes, fractional atomic coordinates for the unit cell of the TPDA-TPB COF with AA stacking, TGA of the TPDA-TPB COF, BET linear and linear fitting plot derived from N_2_ sorption of the TPDA-TPB COF, mass-normalized time-dependent frequency changes upon adsorption of vaporized HCOOH (53 ppm) by the TPDA-TPB COF and a Nafion-coated bare gold electrode of the QCM sensor, mass-normalized frequency variations recorded by the TPDA-TPB COF-modified QCM sensor following being subjected to a formic acid (53 ppm) under different degrees of humidity, analytical characteristic parameters for the sensing activity of the TPDA-TPB COF toward vaporized formic acid using the QCM sensor technique, FTIR spectra of TPDA-TPB before and after 2 months of formic acid detection, TEM images of TPDA-TPB after 2 months of formic acid detection, summary of hazardous formic acid vapor sensing performances of various porous materials, and structural information on the optimized structure of the computed COF model (PDF)
