# Supplementary material for: Covalent-Organic-Framework-Modified Quartz Crystal Microbalance Sensor for Selective Detection of Hazardous Formic Acid
Source: ACS Appl Mater Interfaces. 2024 May 28;16(23):30408–20. doi: 10.1021/acsami.4c04630 (PMC11181274; doi:10.1021/acsami.4c04630)
Supplement: Supplementary file 1 — am4c04630_si_001.pdf [file am4c04630_si_001.pdf]

Supporting Information

Covalent-Organic-Framework-Modified Quartz Crystal Microbalance Sensor for Selective Detection of Hazardous Formic Acid

Lamiaa Reda Ahmed,[a,b] Johann Lüder,[a,c] Cheng-Hsin Chuang,[b] Ahmed F. M. EL-Mahdy,[a*]*

[a]Department of Materials and Optoelectronic Science, National Sun Yat-Sen University, Kaohsiung, 80424, Taiwan

[b]Institute of Medical Science and Technology, National Sun Yat-sen University, Kaohsiung 804201, Taiwan

[c] Center for Theoretical and Computational Physics, National Sun Yat-Sen University, Kaohsiung, 80424, Taiwan

*To whom correspondence should be addressed

E-mail: *chchuang@imst.nsysu.edu.tw*

E-mail: ahmedelmahdy@mail.nsysu.edu.tw

Section	Content	Page No.
**S1**	**Materials**	**S3**
**S2**	**Characterization**	**S3**
**S3**	**Synthetic procedures**	**S4**
**S4**	**FTIR and NMR spectral profiles of monomers**	**S7**
**S5**	**PXRD and BET parameters of the synthesized TPDA-TPB COF**	**S10**
**S6**	**Structural modeling and fractional atomic coordinates for TPDA-TPB COF structure**	**S10**
**S7**	**TGA analysis**	**S12**
**S8**	**BET linear and linear fitting plot**	**S12**
**S9**	**Quartz crystal microbalance gas sensor set up**	**S13**
**S10**	**Information on the computational model**	**S17**

S1. Materials

All chemicals were obtained of analytical grade and were used as received without further purification. Tetrakis(triphenylphosphine)palladium(0) (99%), and palladium on activated carbon (10% Pd/C) were obtained from Acros. 1,2,4,5-Tetrabromobenzene (97.0%) and 4-formylphenylboronic acid (95.0%) were purchased from Sigma–Aldrich. Hydrazine monohydrate (≥98%), and 1-fluoro- 4-nitrobenzene (99%) were obtained from Alfa Aesar. Methanol (MeOH), ethanol (EtOH), tetrahydrofuran (THF), 1,4-dioxane, acetone, and dichloromethane (DCM) were obtained in analytical grade, and were purchased from J.T. Baker.

S2. Characterization

Proton and carbon nuclear magnetic resonance (^{1}H and ^{13}C NMR) spectra were recorded using an INOVA 500 instrument with DMSO-d_6 as solvent and tetramethylsilane (TMS) as the external standard. Chemical shifts are provided in parts per million (ppm).

S3. Synthetic procedures

N^1,N^1,N^4,N^4-Tetrakis(4-aminophenyl)-*p*-phenylenediamine (TPDA-4NH₂)

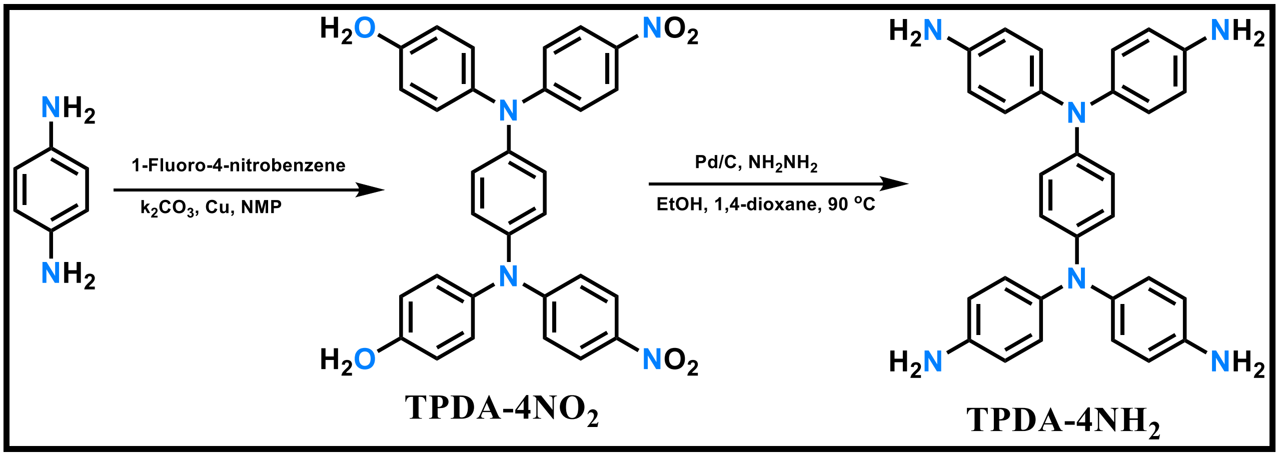

Scheme S1. Synthesis of TPPDA-2NH₂.

***N*¹,*N*¹,*N*⁴,*N*⁴-Tetrakis(4-nitrophenyl)-*p*-phenylenediamine (TPDA-4NO₂):** *p*-Phenylenediamine (5.00 g, 46.0 mmol), 1-fluoro-4-nitrobenzene (39.1 g, 277 mmol), and K_2CO_3 (115 g, 832 mmol) were dissolved in 1-methyl-2-pyrrolidone (60 mL). The mixture was stirred while heating under reflux for 72 h and then cooled to ambient temperature to obtain crystals, which were washed sequentially with 1-methyl-2-pyrrolidone and water and then dried in air to obtain a solid (26.3 g, 96%). M.p.: 300 °C. Mass spectrum: *m*/*z* 592.

***N*¹,*N*¹,*N*⁴,*N*⁴-Tetrakis(4-aminophenyl)-*p*-phenylenediamine (TPDA-4NH₂):** In a 100-mL two-neck round-bottom flask equipped with a stirring bar, TPDA-4NO_2 (2.00 g, 3.37 mmol) and 10% Pd/C (0.20 g) were suspended in EtOH (20 mL) and 1,4-dioxane (40 mL) under a N_2 atmosphere. The suspension was heated at 90 °C for 15 min before hydrazine monohydrate (5.5 mL) was added slowly. The mixture was stirred at 90 °C for 36 h, and then it was filtered to remove the Pd/C. The filtrate was cooled, giving greenish crystals, which were filtered off and dried under vacuum at 70 °C to obtain TPPDA-4NH_2 (0.8 g, 50%). FTIR (powder): 3455, 3431, 3334, 3063–2836, 1625, 1500, 1256, 824 cm^{-1} (**Figure S1**). ^{1}H NMR (DMSO-d_6, 25 °C, 500 MHz): 4.81 (br s, 4NH_2, 8H),

6.46 (d, J = 8.50 Hz, 12H), 6.68 (d, J = 8.50 Hz, 8H) (**Figure S2**). ^{13}C NMR (DMSO-d_6, 25 °C, 125 MHz): 145.17, 142.57, 138.05, 126.49, 120.96, 115.44 (**Figure S3**).

4',5'-bis(4-formylphenyl)-[1,1':2',1''-terphenyl]-4,4''-dicarbaldehyde (TPB-4CHO)

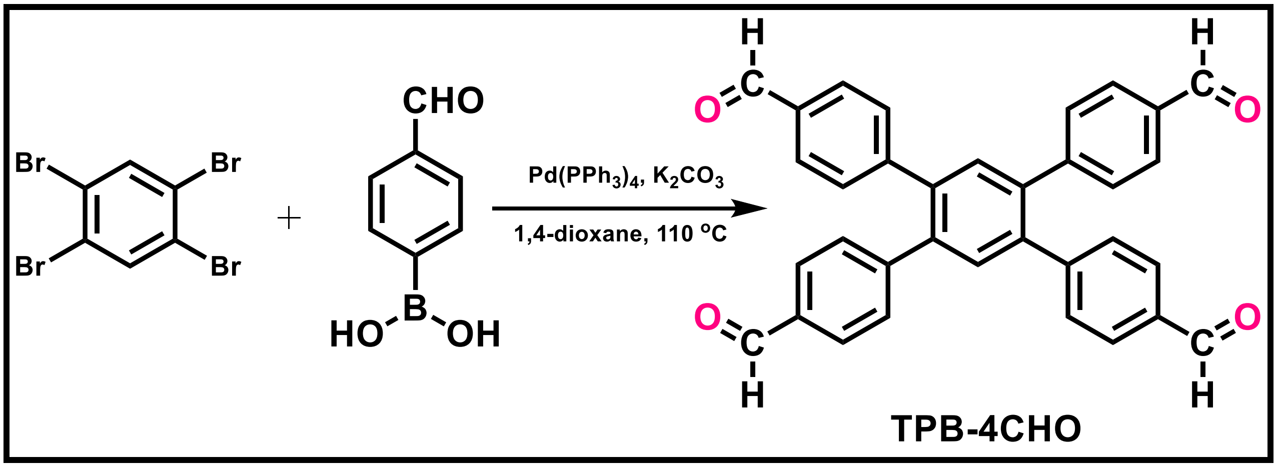

Scheme S2. Synthesis of TPB-4CHO.

4',5'-bis(4-formylphenyl)-[1,1':2',1''-terphenyl]-4,4''-dicarbaldehyde (TPB-4CHO): A 100-mL round-bottom flask was charged with 1,2,4,5-tetrabromobenzene (0.7 g, 1.77 mmol), 4-formylphenylboronic acid (2.13 g, 14.2 mmol), tetrakis(triphenylphosphine)palladium(0) (105 mg, 0.090 mmol), and K_2CO_3 (2.5 g, 18.09 mmol). The solid mixture was evacuated under low pressure for 15 min. Dioxane (40 mL) and water (8 mL) were added, and then the mixture was heated at 110 °C for 72 h under N_2. After the consumption of 1,2,4,5-tetrabromobenzene, the mixture was cooled to room temperature and poured into ice water to produce a white precipitate. The solid was filtered off and washed several times with water and methanol. The isolated solid TPB-4CHO was used without further purification (87%). FTIR (powder): 2814–2723, 1701, 1602, 1389, 1210, 1169, 821 824 cm^{-1} (**Figure S4**). ^{1}H NMR (500 MHz, $CDCl_3$) δ (ppm): 10.00 (s, 4H), 7.80 (d, J = 8 Hz, 8H), 7.60 (s, 2H), 7.36 (d, J = 8 Hz, 8H) (**Figure S5**). ^{13}C NMR (125 MHz, $CDCl_3$) δ (ppm): 192.47, 146.83, 140.21, 135.70, 133.05, 130.64 (**Figure S6**).

TPDA-TPB COF

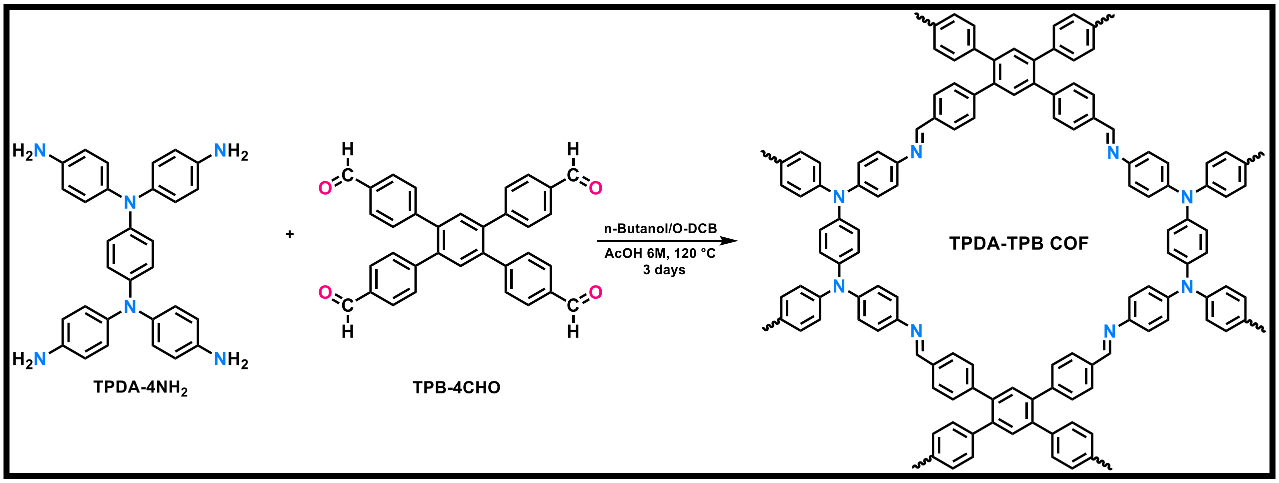

Scheme S3. Synthesis of TPDA-TPB COF.

TPDA-4NH$_2$ (100 mg, 0.21 mmol) and TPB-4CHO (104.64 mg, 0.21 mmol) were placed in a 25-mL tube. They were then mixed with a solution containing equal parts *o*-dichlorobenzene and *n*-butanol (1:1, 10 mL), as well as a 6 M aqueous solution of acetic acid (1.0 mL). The tube underwent multiple freeze/pump/thaw cycles to remove gas and was subsequently sealed using flame and heated to a temperature of 120 °C. Following a period of 72 hours, a solid substance was produced. This solid was then subjected to filtration and underwent many rounds of washing using acetone and tetrahydrofuran (THF) in a sequential manner. Achieved a 91% yield of TPDA-TPB COF using vacuum drying at 120 °C for 24 hours.

S4. FTIR and NMR spectral profiles of monomers

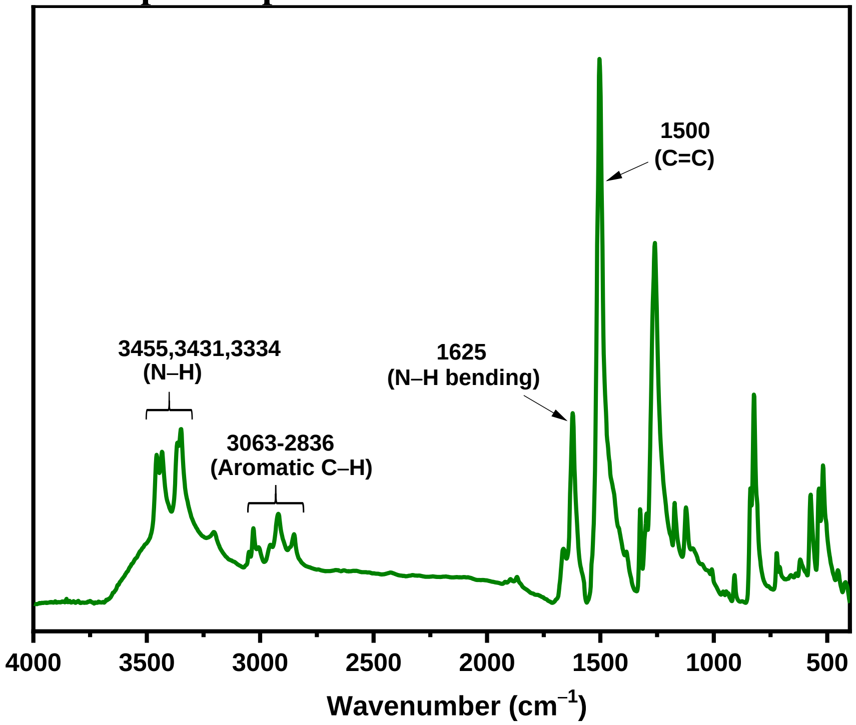

Figure S1. FT-IR spectrum of TPDA-4NH2.

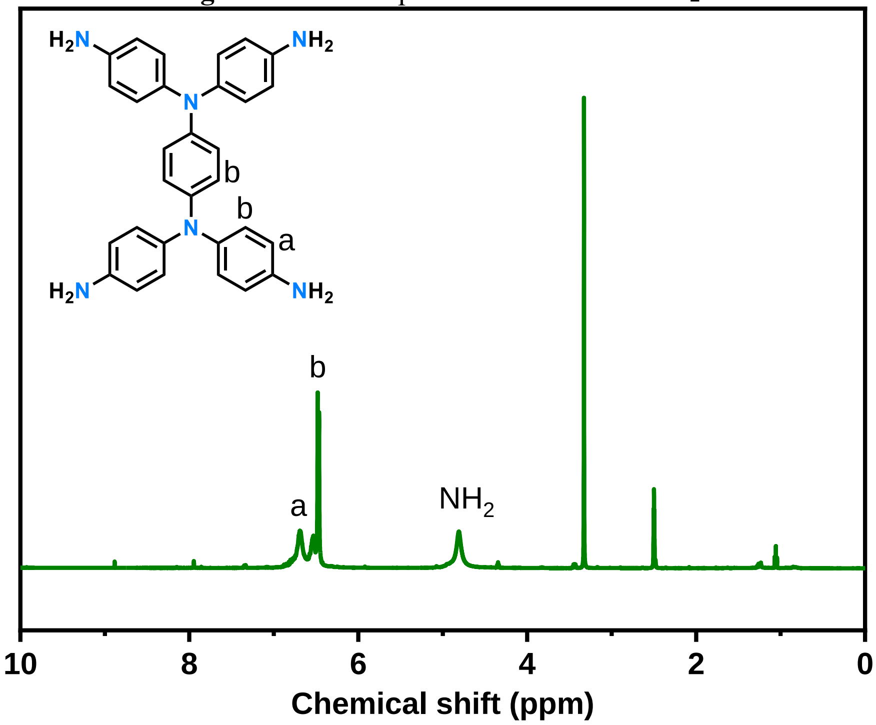

Figure S2. ^{1}H NMR spectrum of TPDA-4NH2.

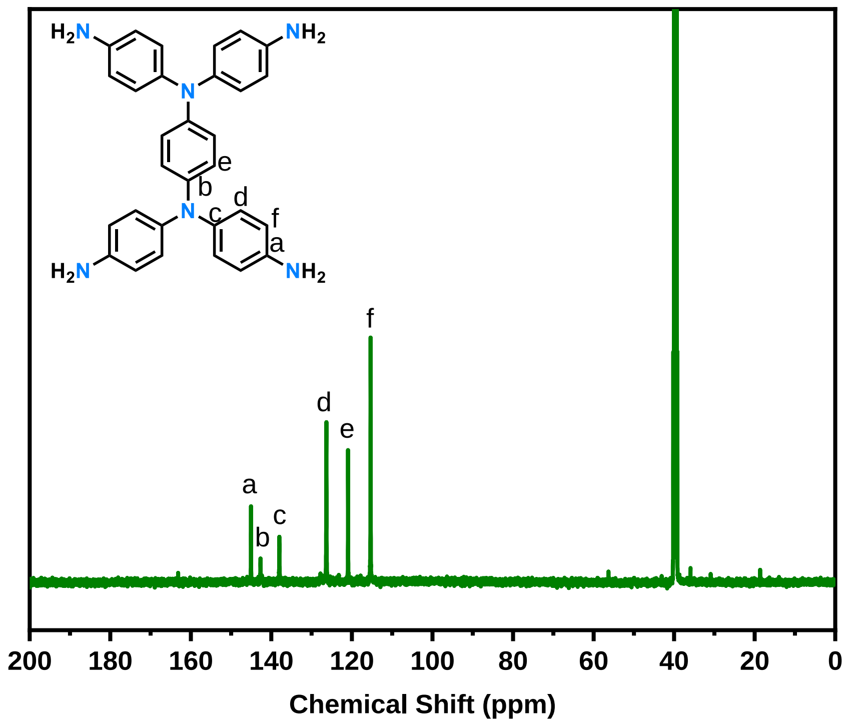

Figure S3. ^{13}C NMR spectrum of TPDA-4NH_2.

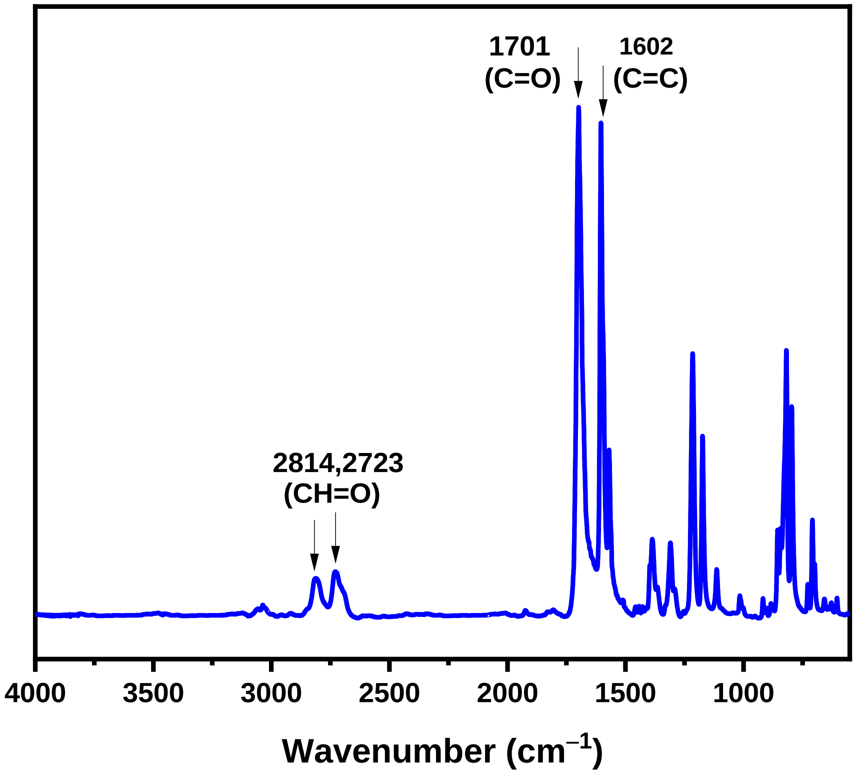

Figure S4. FT-IR spectrum of TPB-4CHO.

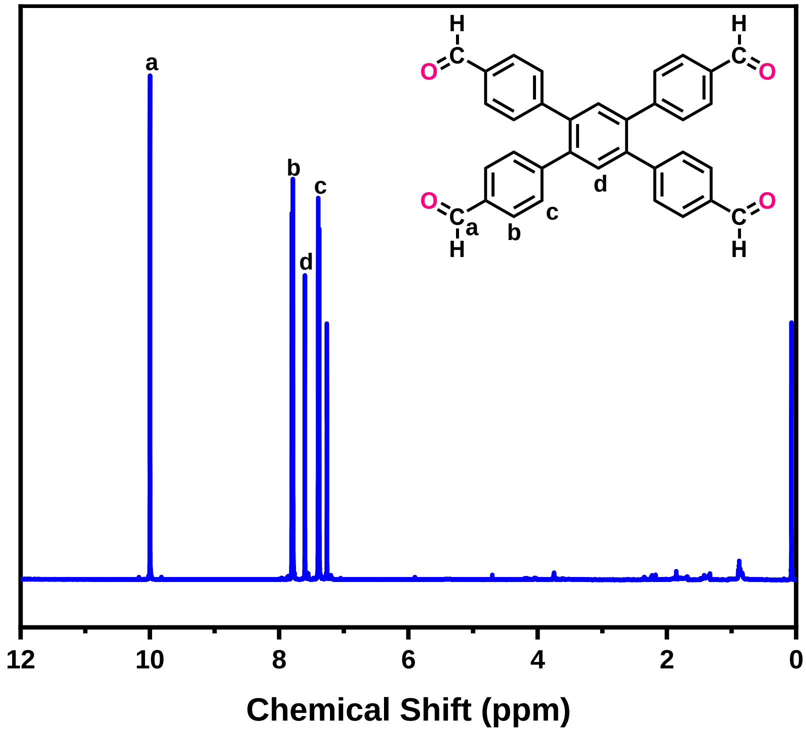

Figure S5. ^{1}H NMR spectrum of TPB-4CHO.

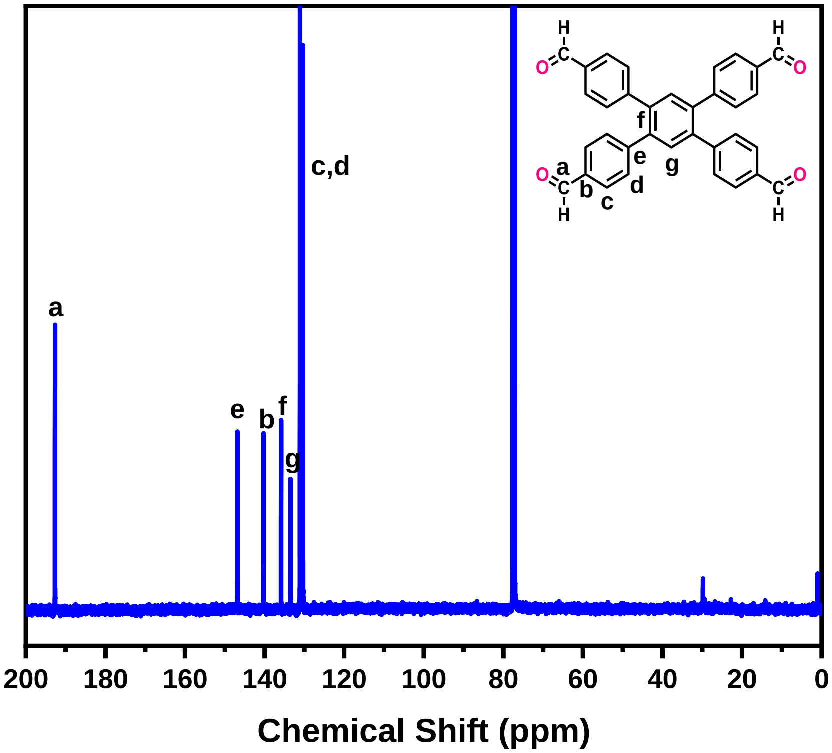

Figure S6. ^{13}C NMR spectrum of TPB-4CHO.

S5. PXRD and BET parameters of the synthesized TPDA-TPB COF.

Table S1. PXRD and BET parameters of the synthesized TPDA-TPB COF.

COF	S_{BET} (m^2 g^{-1})	d_{100} (nm)	Pore size (nm)	Pore volume (cm^3 g^{-1})	Interlayer Distance (Å)
TPDA-TPB	852	1.50	1.38	0.89	3.47

S6 Structural modeling and fractional atomic coordinates for TPDA-TPB COF structure

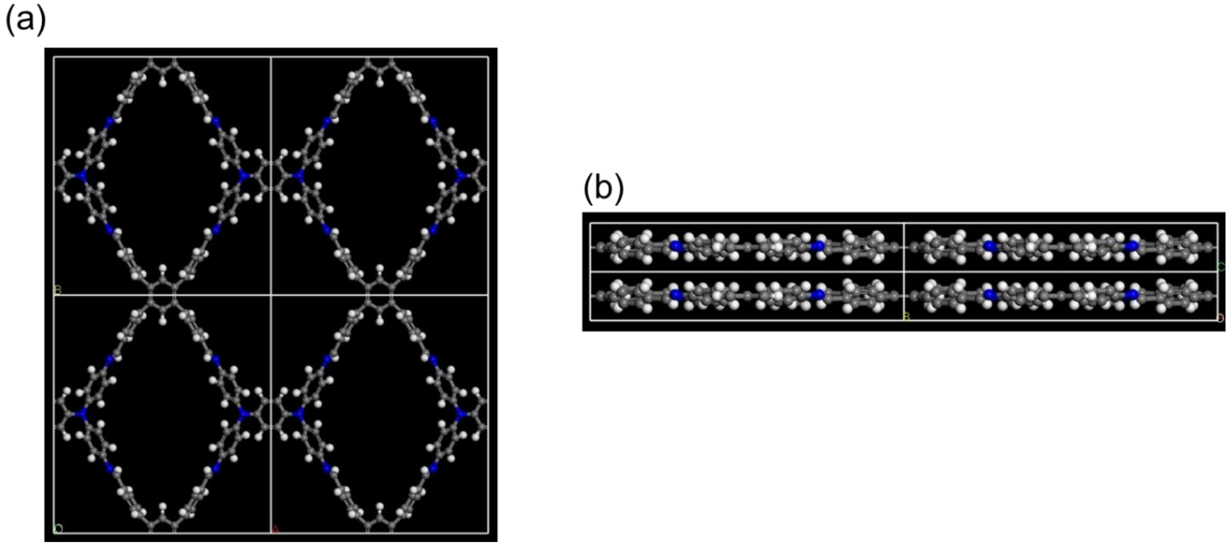

Figure S7. Crystalline structure for TPDA-TPB COF based on AA–eclipsed stacking model along (a) *c* axis and (b) *a* axis.

Table S2. Fractional atomic coordinates for the unit cell of TPDA-TPB COF with AA-stacking.

Sample Name : TPDA-TPB COF							
Space Group : P 1 2/M 1							
a = 20.60 Å, *b* = 22.60 Å, *c* = 3.51 Å, *α* = 90°, *α* = 90°, γ = 90°							
R_{wp}= 9.43% R_p= 6.52%							
Atom	*x/a*	*y/b*	*z/c*	Atom	*x/a*	*y/b*	*z/c*
C1	0.03098	0.55391	0.49981	C16	0.06399	0.5	0.50028
C2	0.16146	0.55769	0.50199	C17	0.5	0.93812	0.5
C3	0.15163	0.39995	0.60471	N18	0.25594	0.27053	0.5293
C4	0.18252	0.34464	0.61143	N19	0.12889	0.5	0.49707
C5	0.22449	0.32819	0.51539	H20	0.05935	0.59722	0.49908
C6	0.2348	0.37039	0.41199	H21	0.11609	0.41164	0.68774
C7	0.20406	0.42634	0.4059	H22	0.17199	0.31359	0.69848
C8	0.28849	0.24351	0.43901	H23	0.27009	0.35839	0.32872
C9	0.44506	0.96732	0.49145	H24	0.21524	0.45788	0.3201
C10	0.60659	0.92431	0.52294	H25	0.29416	0.26408	0.33577
C11	0.61498	0.89367	0.64472	H26	0.59191	0.91256	0.73888
C12	0.65018	0.84136	0.65463	H27	0.65597	0.81922	0.75614
C13	0.67829	0.81581	0.54312	H28	0.69395	0.82727	0.3266
C14	0.67046	0.84617	0.42031	H29	0.62867	0.92088	0.30892
C15	0.63473	0.89878	0.41039	H30	0.5	0.88769	0.5

S7. TGA analysis

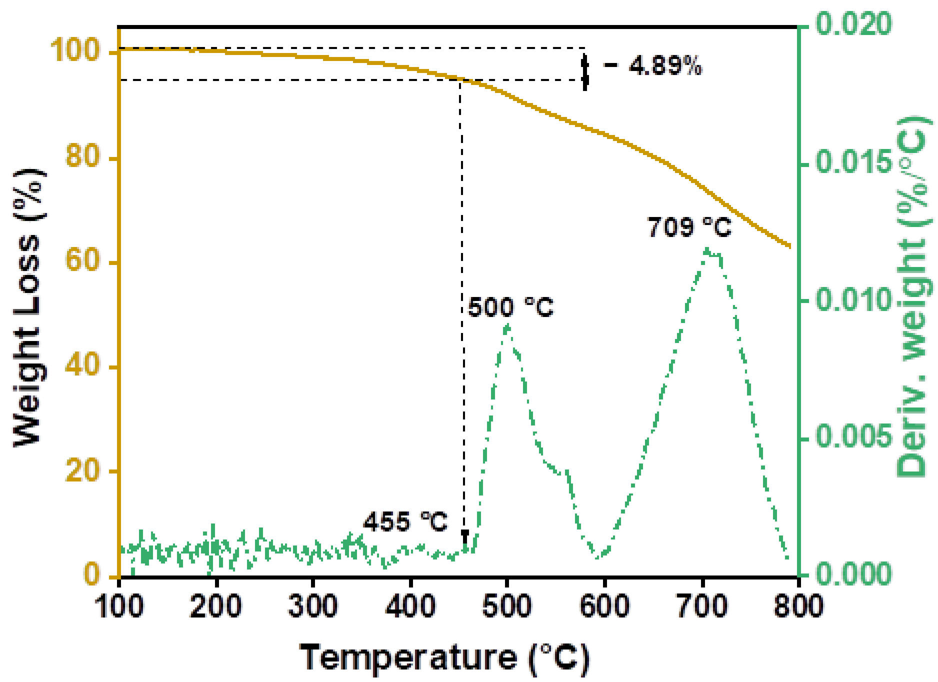

Figure S8. TGA analysis of TPDA-TPB COF.

S8. BET linear and linear fitting plot

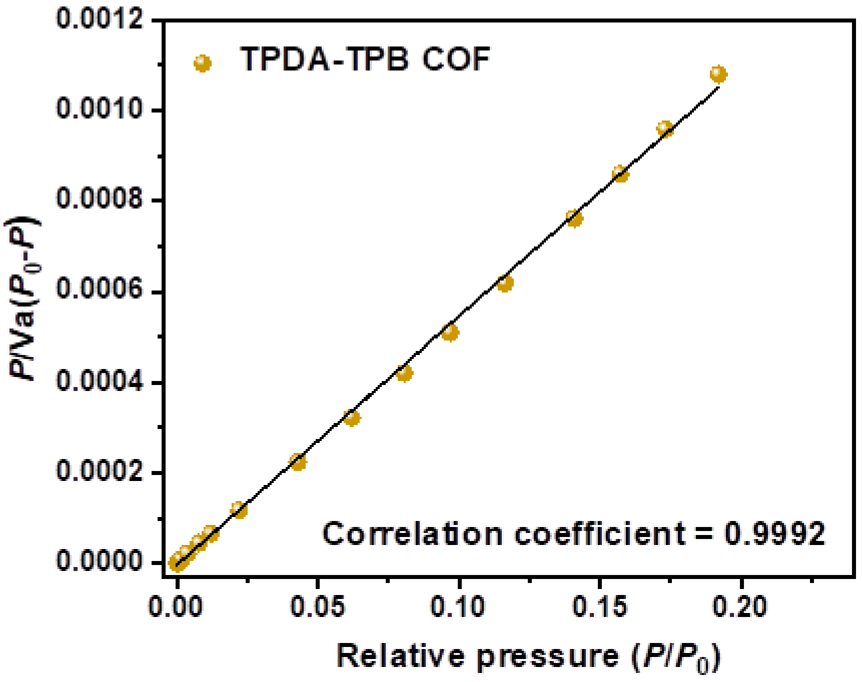

Figure S9. BET linear and linear fitting plots derived from N_2 sorption of TPDA-TPB COF.

S9. Quartz crystal microbalance gas sensor

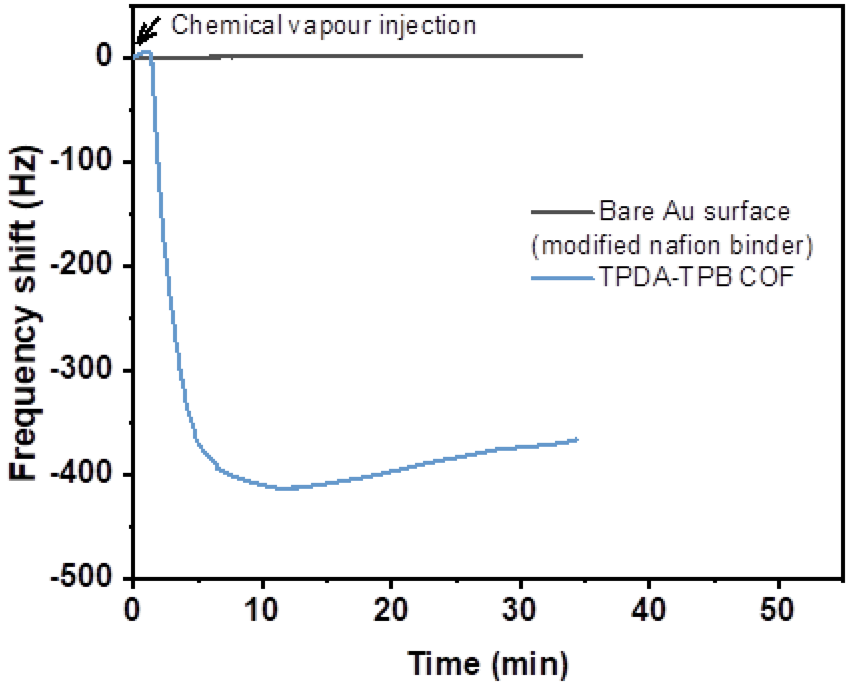

Figure S10. Mass-normalized time-dependent frequency changes upon adsorption of vaporized HCOOH (53 ppm) by TPDA-TPB COF and Nafion-coated bare Au electrode of the QCM sensor.

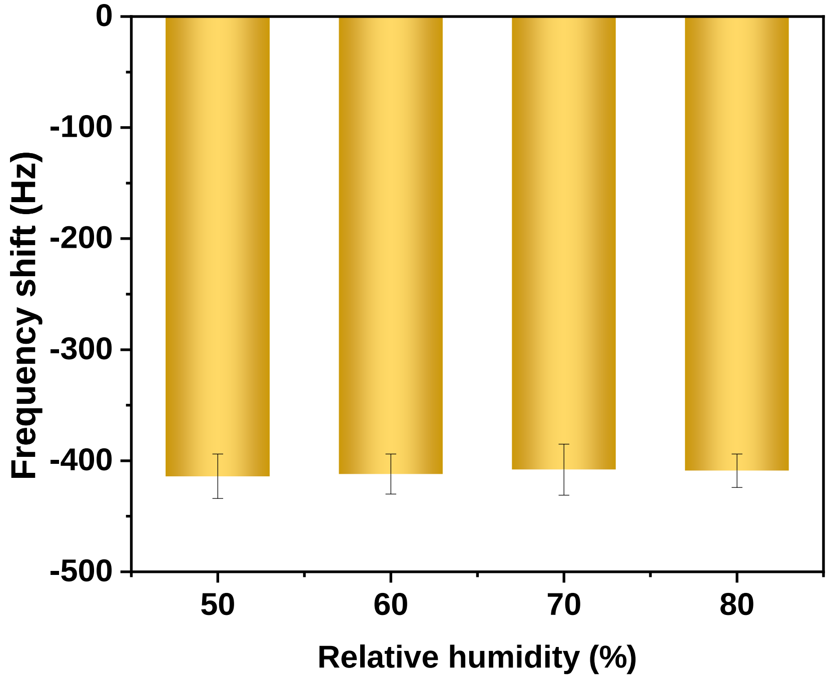

Figure S11. The mass-normalized frequency variations recorded by the TPDA-TPB COF -modified QCM sensor following being subjected to a formic acid (53 ppm) under different degrees of humidity.

Table S3. Analytical characteristic parameters for sensing activity of the TPDA-TPB COF towards vaporized formic acid using the QCM sensor technique.

COF	ΔF (Hz)	Sensitivity (Hz ppm^{-1})	Limit of detection (LOD, ppm)	R^{2a}	RSD[b] (%) (n = 4)
TPDA-TPB	418.40 ± 20	7.75	1.18	0.9993	3.08

[a] Correlation coefficient

[b] Relative standard deviation

n: Number of formic acid injections.

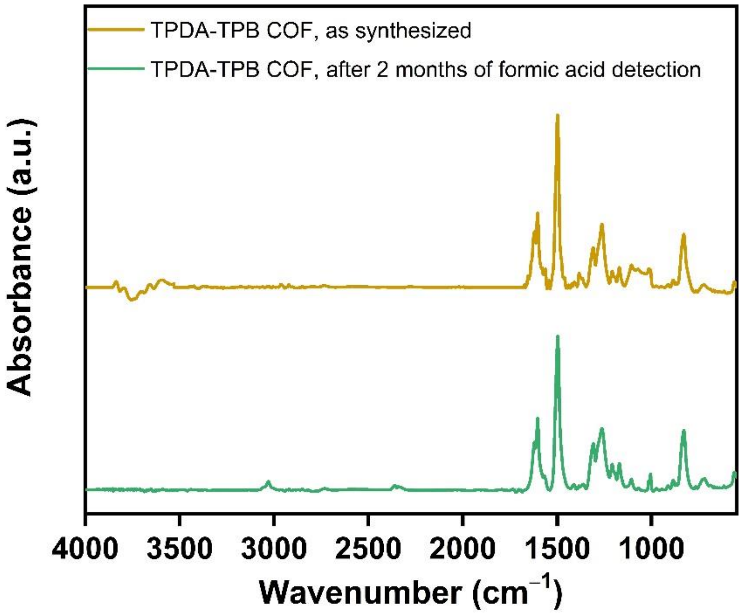

Figure S12. FTIR spectra of TPDA-TPB before and after 2 months of the formic acid detection.

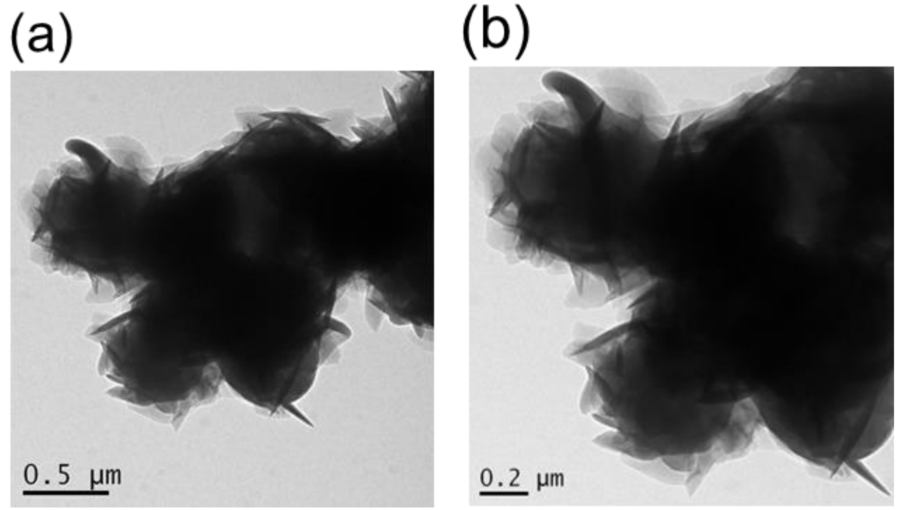

Figure S13. TEM images of TPDA-TPB after 2 months of the formic acid detection.

Table S4. Summary of hazardous formic acid vapor sensing performances of various porous materials.

Materials	Surface area ($m^2\ g^{-1}$)	Sensing technique	Sensitivity ($Hz\ ppm^{-1}$)	LOD (ppm)	Ref.
multi-walled carbon nanotubes /polyaniline (MWCNTs/PANI)	--	QCM sensor	38.51	--	*Res. Chem. Intermed.* **2023**, *49* (9), 3893-3907
multi-walled carbon nanotubes	--	QCM sensor	--	0.77	*Environ. Technol.* **2023**, *44* (6), 751-761.
Carbon nitride	635	QCM sensor	--	--	*J. Mater. Chem. A*, **2013**, *1*, 2913−2920.
Ph-g-C_3N_4 nanoflakes	306	QCM sensor	128.99	0.08	*ACS Appl. Mater. Interfaces* **2021**, *13* (41), 48595-48610.
Polyaniline	--	QCM sensor	--	0.53	*Water, Air, Soil Pollut.* **2012**, *223*, 1275−1280.
4-Ethyl-3-thiosemicarbazide	--	QCM sensor	29.7	0.17	*Analyst* **1993**, *118*, 175−178.
2,6-diacetylpyridine	--	QCM sensor	10.9	0.33	*Analyst* **1993**, *118*, 175−178.
Polyoxyethylene bis (amine)	--	QCM sensor	0.67	7.2	*J. Anal. Chem.* **2001**, *370*, 393−398.
Single-walled carbon nanotubes (CNTs) and nickel bis(ortho-diiminosemiquinonate)	--	Chemiresistor sensor	--	0.083	*ACS Sens.* **2018**, *3*, 569−573.
metal-organic framework (MOF) thin film	--	Impedance sensor	--	11.04	*Micropor. Mesopor. Mat.* **2023**, *360*, 112722
Cobalt(II)-organic frameworks {[Co3(p-CPhHIDC)$_2$(4,4′-bipy)(H_2O)]·2 H_2O}n	8.44	Impedance sensor	--	35	*Chem. - Eur. J.* **2019**, *25*, 14108−14116
{[Co3(p-CPhHIDC)$_2$ (bpe)(H_2O)]·3 H_2O}n	6.17	Impedance sensor	--	70	*Chem. - Eur. J.* **2019**, *25*, 14108−14116
3D isostructural MOFs ZZU-1	9.8	Impedance sensor	--	552.4	*Chem. Asian J.* **2020**, *15*, 182−190
3D isostructural MOFs ZZU-2	12.8	Impedance sensor	--	920.7	*Chem. Asian J.* **2020**, *15*, 182−190
FDH, NAD+ and Meldola's blue immobilized in glycerol	--	Amperometric biosensor	--	1.0	*Sens. Actuators, B* **2000**, *70*, 182−187.
b(III) Functionalized Metal-Organic Framework	--	Fluorescence sensor	--	2.1	*Molecules,* **2022**, *27,* 8702.
SnO_2 quantum dot sensitized $LaFeO_3$ (LSO-2)	21.47	conductometric sensor	--	1.0	*Sens. Actuators B Chem.* **2023**, *379,* 133198.
zirconium-organic xerogel (Zr/Fum-xerogel-0.04)	486	conductometric sensor	--	150	*J. Colloid Interface Sci.* **2022**, *607,* 181−191.
**TPDA-TPB COF**	**852**	**QCM sensor**	**7.75**	**1.18**	**This work**

S10. Information on the computational model

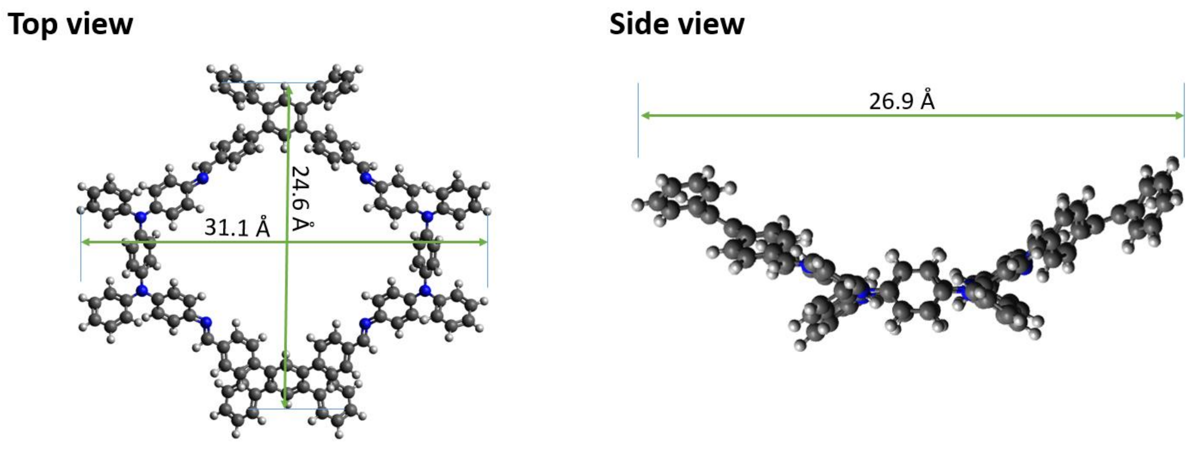

Figure S14. Structural information on the optimized structure of computed COF model. Color code: gray: carbon, white: hydrogen, blue nitrogen atoms.
